# Up-Regulated Expression of Extracellular Matrix Remodeling Genes in Phagocytically Challenged Trabecular Meshwork Cells

**DOI:** 10.1371/journal.pone.0034792

**Published:** 2012-04-18

**Authors:** Kristine M. Porter, David L. Epstein, Paloma B. Liton

**Affiliations:** Duke University, Department of Ophthalmology, Durham, North Carolina, United States of America; University of Turin, Italy

## Abstract

**Background:**

Cells in the trabecular meshwork (TM), the tissue responsible for draining aqueous humor out of the eye, are known to be highly phagocytic. Phagocytic function in TM cells is thought to play an important role in the normal functioning of the outflow pathway. Dysfunction of phagocytosis could lead to abnormalities of outflow resistance and increased intraocular pressure (IOP). However, the molecular mechanisms triggered by phagocytosis in TM cells are completely unknown.

**Methodology/Principal Findings:**

Gene expression profile analysis of human TM cells phagocytically challenged to E. coli or pigment under physiological and oxidative stress environment were performed using Affymetrix U133 plus 2.0 array and analyzed with Genespring GX. Despite the differential biological response elicited by E. coli and pigment particles, a number of genes, including MMP1, MMP3, TNFSF11, DIO2, KYNU, and KCCN2 showed differential expression with both phagocytic ligands in all conditions. Data was confirmed by qPCR in both human and porcine TM cells. Metacore pathway analysis and the usage of recombinant adenovirus encoding the dominant negative mutant of IkB identified NF-κB as a transcription factor mediating the up-regulation of at least MMP1 and MMP3 in TM cells with phagocytosis. In-gel zymography demonstrated increased collagenolytic and caseinolytic activities in the culture media of TM cells challenge to E. coli. In addition, collagenolytic I activity was further confirmed using the self-quenched fluorescent substrate DQ-Collagen I.

**Conclusions/Significance:**

Here we report for the first time the differential gene expression profile of TM cells phagocytically challenged with either E. coli or pigment. Our data indicate a potential role of phagocytosis in outflow pathway tissue homeostasis through the up-regulation and/or proteolytic activation of extracellular matrix remodeling genes.

## Introduction

Glaucoma is a group of blinding disorders affecting more than 70 million people worldwide, which is characterized by irreversible damage to the optic nerve. The major risk factor for developing glaucoma is elevated intraocular pressure (IOP), which results from the increased resistance to aqueous humor outflow through the trabecular meshwork (TM) conventional outflow pathway [1], [2].

The TM is a tiny tissue located in the anterior segment of the eye between the cornea and the sclera. It is structured into three differentiated layers through which the aqueous humor must pass before leaving the eye: the inner uveal meshwork, the corneoscleral meshwork and the juxtacanalinular tissue (JCT). The uveal and the corneoscleral meshworks are composed of sheets of connective tissue beams lined by TM endothelial cells. The beams attach to each other in several layers forming a porous filter-like structure [3], [4].

Trabecular meshwork cells lining the beams are known to be able to avidly phagocyte particulate material and debris in vitro and in vivo [5]–[11]. Because of this phagocytic activity, the meshwork has been suggested to function in vivo as a self-cleaning filter able to keep the drainage channels free of obstructive material or debris, which otherwise might block the flow of aqueous humor [6]. Thereby, phagocytosis is thought to have an important role in the normal functioning of the outflow pathway. Abnormalities in phagocytosis have been postulated to contribute to the development of certain types of glaucoma, in particular in exfoliative, pigmentary, phagolytic, and other obstructive glaucomas [12]–[14].

While a number of studies have shown the detachment of TM cells from the trabecular beams following phagocytosis in vivo and in vitro [5], [7]–[9], [15], as well as short-term loss in cell-matrix cohesiveness cell culture conditions [16], [17], the molecular mechanisms encompassing such events have yet to be clarified.

Here we report for the first time the transcriptome profile of TM cells phagocytically challenged with either E. coli or pigment under physiological and oxidative stress conditions. Our data demonstrate the upregulation of metalloproteinases and extracellular matrix (ECM) remodeling upon phagocytosis in TM cells.

## Results

### Differential Gene Expression Profile of Human TM Cells Phagocytically Challenged Under Physiological Conditions

**Figure 1:**
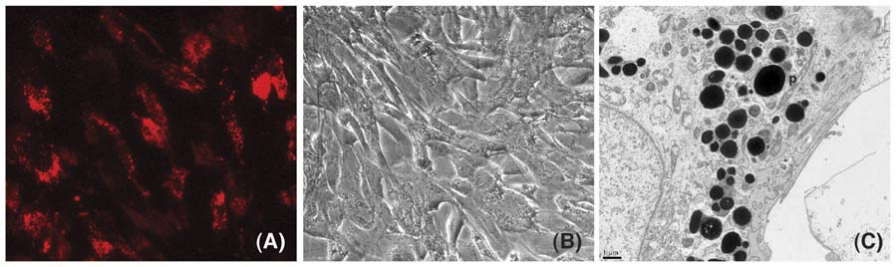
Phagocytic activity in TM cells. (A) Fluorescence microcopy image of human TM cells phagocytically challenged with pHRodo-labeled E. coli (1×10 ^6^ particles/mL). Note the puncta red staining indicating the presence of engulfed E. coli particles. (B) Light microscopy image of human TM cells phagocytically challenged to pigment particles (1×10 ^6^ particles/mL). (C) Electron micrograph image of human TM cells phagocytically challenged to pigment particles. Note the presence of numerous engulfed pigment particles (p) contained in membrane-bound organelles.

Confluent cultures of human TM cells were grown for two weeks under physiological conditions and then challenged for three days, time-point at which the phagocytic capacity of TM cells is peaked [17], to saturated doses of either pHRodo-labeled E. coli or pigment. Changes in gene expression induced by phagocytosis were evaluated by gene array analysis using Affymetrix Human Genome U133 Plus 2.0 chips. Comparative analysis showed 1190 and 728 genes significantly up-regulated and down-regulated, respectively, more than 1.5-fold in TM cells phagocytically challenged to E. coli. A complete list of the genes with differential expression greater than two is included as Supporting Information (Table S1, Table S2). Phagocytosis of pigment particles elicited a much lesser biological response. Only 26 and 14 genes were found to be significantly up-regulated (Table S3) and down-regulated (Table S4) more than 1.5 fold, respectively, in TM cells challenged to pigment. As shown in Figure 1, more than 90% of the cells in the culture were phagocytic cells. Electron micrographs confirmed the presence of engulfed pigment particles within the cells (Figure 1).

Table 1 lists the genes (21 genes), whose expression was consistently up-regulated with phagocytosis of both E. coli and pigment. These could largely be clustered into two different categories: (i) genes involved in the immune response such as chemokine ligands (CXCL5, CXCL6), interleukins (IL32, IL33), complement system (C3), and TNFSF11; and (ii) genes involved in cell adhesion and extracellular matrix (ECM) remodeling (MMP1, MMP3, LAMC2, TFPI2). Genes consistently down-regulated with phagocytosis were more heterogeneous (10 genes, Table 2) and could not be clustered into known biological categories.

**Table 1:** Genes Significantly Upregulated (>1.5 fold, p<0.05) in HTM Cells Phagocytically Challenged Under Physiological Conditions.

Gene Title	Gene Symbol	UniGene ID	*E. coli*		*Pigment*		Chromosomal Location
			Fold	pValue	Fold	pValue	
acyl-CoA synthetase long-chain family member 4	ACSL4	Hs.268785	3.01	0.009	1.49	0.017	chrXq22.3-q23
calcium binding protein 1	CABP1	Hs.458482	2.62	0.022	1.46	0.039	chr12q24.31
chemokine (C-X-C motif) ligand 5	CXCL5	Hs.89714	54.52	0.002	1.46	0.004	chr4q12-q13
chemokine (C-X-C motif) ligand 6 (granulocyte chemotactic protein 2)	CXCL6	Hs.164021	5.30	0.008	1.51	0.014	chr4q21
chromosome 6 open reading frame 58	C6orf58	Hs.226268	1.64	0.021	1.47	0.038	chr6q22.33
complement component 3	C3	Hs.529053	10.59	0.003	1.48	0.006	chr19p13.3-p13.2
deiodinase, iodothyronine, type II	DIO2	Hs.202354	3.83	0.004	1.92	0.006	chr14q24.2-q24.3
ets homologous factor	EHF	Hs.653859	3.05	0.019	1.49	0.034	chr11p12
interleukin 32	IL32	Hs.943	6.20	0.004	1.56	0.006	chr16p13.3
interleukin 33	IL33	Hs.348390	22.50	0.002	2.16	0.004	chr9p24.1
kynureninase (L-kynurenine hydrolase)	KYNU	Hs.470126	7.34	0.007	1.75	0.013	chr2q22.2
laminin, gamma 2	LAMC2	Hs.591484	1.97	0.026	1.47	0.046	chr1q25-q31
matrix metallopeptidase 1 (interstitial collagenase)	MMP1	Hs.83169	4.61	0.003	1.98	0.006	chr11q22.3
matrix metallopeptidase 3 (stromelysin 1, progelatinase)	MMP3	Hs.375129	11.20	0.004	2.09	0.006	chr11q22.3
six transmembrane epithelial antigen of the prostate 1	STEAP1	Hs.61635	6.03	0.003	1.53	0.005	chr7q21
solute carrier family 39 (zinc transporter), member 8	SLC39A8	Hs.288034	11.27	0.003	1.50	0.006	chr4q22-q24
stanniocalcin 1	STC1	Hs.25590	12.64	0.002	1.51	0.003	chr8p21-p11.2
superoxide dismutase 2, mitochondrial	SOD2	Hs.487046	24.05	0.004	1.56	0.006	chr6q25.3
tissue factor pathway inhibitor 2	TFPI2	Hs.438231	8.15	0.007	1.81	0.012	chr7q22
tumor necrosis factor (ligand) superfamily, member 11	TNFSF11	Hs.333791	2.72	0.005	1.87	0.009	chr13q14
UDP-N-acetyl-alpha-D-galactosamine∶polypeptide N-acetylgalactosaminyltransferase-like 2	GALNTL2	Hs.411308	1.54	0.012	1.59	0.022	chr3p24.3

**Table 2:** Genes Significantly Downregulated (>1.5 fold, p<0.05) in HTM Cells Phagocytically Challenged Under Physiological Condition.

Gene Title	Gene Symbol	UniGene ID	*E. coli*		*Pigment*		Chromosomal Location
			Fold	pValue	Fold	pValue	
WAP four-disulfide core domain 1	WFDC1	Hs.36688	2.22	0.002	1.58	0.004	chr16q24.3
Tetraspanin 18	TSPAN18	Hs.385634	2.19	0.003	1.77	0.006	chr11p11.2
potassium intermediate/small conductance calcium-activated channel, subfamily N, member 2	KCNN2	Hs.98280	2.00	0.028	1.48	0.050	chr5q22.3
hydroxysteroid (17-beta) dehydrogenase 6 homolog (mouse)	HSD17B6	Hs.524513	2.52	0.013	1.51	0.023	chr12q13
H19, imprinted maternally expressed transcript (non-protein coding)	H19	Hs.533566	4.04	0.002	1.47	0.004	chr11p15.5
fibroblast growth factor receptor 3	FGFR3	Hs.1420	1.58	0.010	1.61	0.019	chr4p16.3
endothelin 3	EDN3	Hs.1408	5.78	0.004	1.46	0.006	chr20q13.2-q13.3
ArfGAP with RhoGAP domain, ankyrin repeat and PH domain 2	ARAP2	Hs.479451	2.27	0.011	1.52	0.020	chr4p14
aquaporin 1 (Colton blood group)	AQP1	Hs.76152	2.99	0.006	1.51	0.012	chr7p14
ankyrin repeat domain 6	ANKRD6	Hs.702213	1.99	0.015	1.82	0.027	chr6q14.2-q16.1

**Figure 2:**
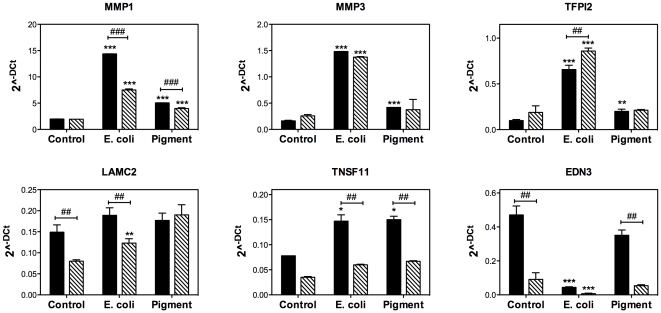
Quantitative real-time PCR confirmation of selected genes with differential expression in phagocytically challenged human TM cells under physiological (black bars) and oxidative stress (stripped bars) conditions. The expression levels were calculated using the formula 2^−ΔCt^, where ΔCt = Ct_gene_−Ct _average housekeeping_. β-Actin, GAPDH, and HPRT1 served as internal standard for normalization. Values represent mean ± SD. (*) compares phagocytically challenged versus control cultures; (^#^) compares oxidatively stressed versus cultures grown under physiological conditions. *, ^#^ p<0.05, **, ^##^ p<0.005, ***, ^###^ p<0.0005 (t-test, n = 3).

**Figure 3:**
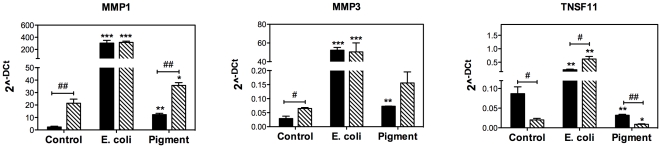
Quantitative real-time PCR confirmation of selected genes with differential expression in phagocytically challenged porcine TM cells under physiological (black bars) and oxidative stress (stripped bars) conditions. The expression levels were calculated using the formula 2^−ΔCt^, where ΔCt = Ct_gene_−Ct _average housekeeping_. β-Actin, GAPDH, and HPRT1 served as internal standard for normalization. Values represent mean ± SD. (*) compares phagocytically challenged versus control cultures; (^#^) compares oxidatively stressed versus cultures grown under physiological conditions. *, ^#^ p<0.05, **, ^##^ p<0.005, ***, ^###^ p<0.0005 (t-test, n = 3).

Changes in gene expression of selected genes were quantified by qPCR (Figure 2, black bars). With the exemption of LAMC2, which showed a slight but non-statistically significant up-regulation, quantitative PCR validated the results obtained from microarray analysis. In addition, differential expression of MMP1, MMP3, and TNFSF11 in response to phagocytic challenge was further confirmed in porcine TM cells (Figure 3, black bars). These changes occurred as early as one day post-challenge in E.coli-treated cells, but later (day 3) in the cultures exposed to pigment (Figure S1). Summary of the expression levels obtained in the qPCR experiments are included as Supporting Information (Table S5).

### Functional Network Analysis of Gene Expression Changes in Phagocytically Challenged human TM Cells

**Figure 4:**
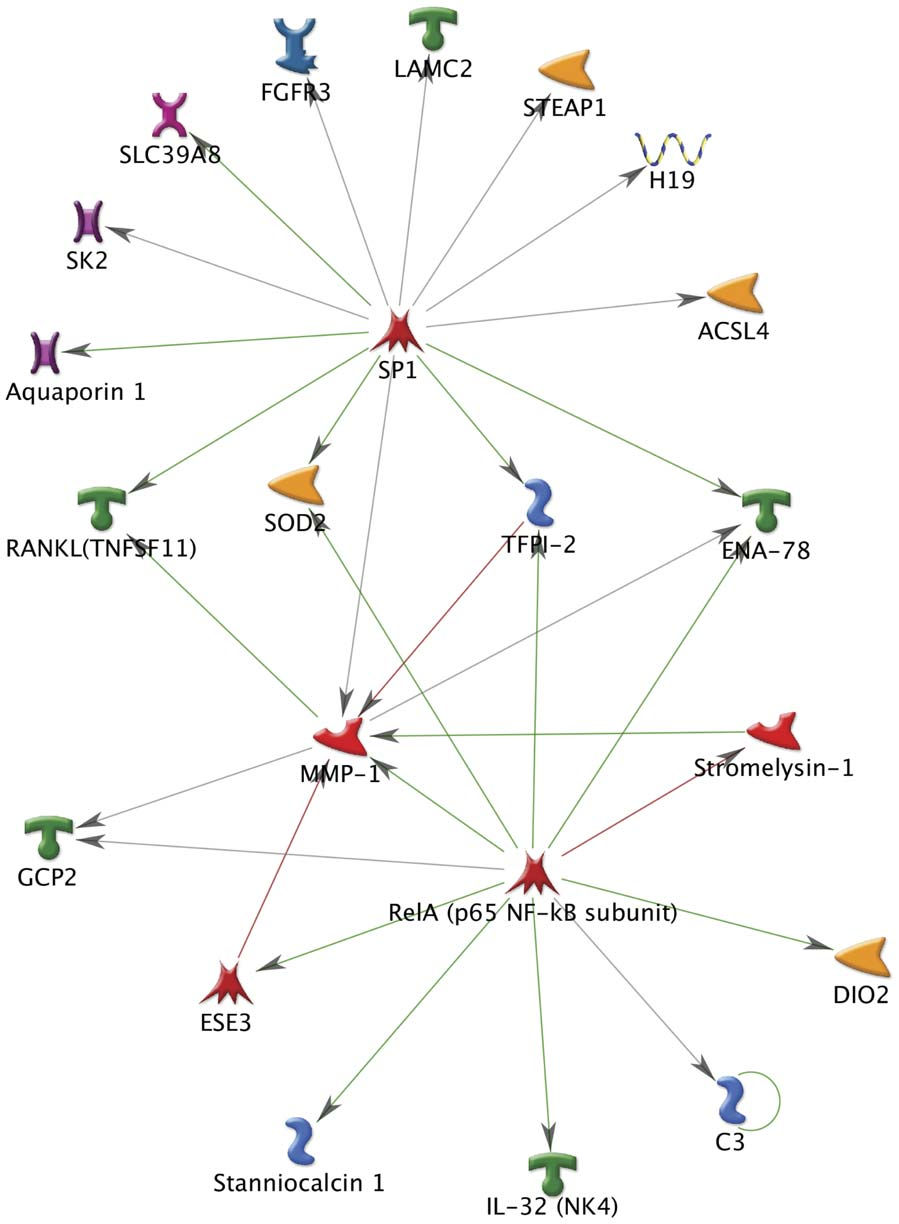
Pathway analysis of the genes showing changes in expression higher than 1.5 fold (p<0.05) in human TM cells phagocitically challenged to E.coli and pigment. NF-kB and SP1 were identified as the transcription factors with the highest ranking in terms of P-value and gene ontology interpretation.

To identify potential pathways and regulatory elements associated with changes in gene expression induced by phagocytosis in human TM cells, gene lists obtained from microarray analysis were further analyzed using the pathway analysis and data mining software MetaCore (GeneGo). Table 3 summarizes the top pathways and networks identified in human TM cells subjected to phagocytic stress. Collectively, functional network analysis of microarray data showed an enrichment in the immune response and cell adhesion/ECM remodeling pathways in phagocytically challenged human TM cells. In addition, SP1 and NF-κB were identified as the transcription factors most likely to be differentially active and responsible for the changes in gene expression upon phagocytosis (Figure 4).

**Table 3:** Top Pathways Identified in Phagocytically Challenged TM Cells Using GeneGo Metacore Analysis.

Name	pValue
Immune response_Complement pathway	3.95E-10
Immune response_IL-17 signaling pathways	3.27E-07
Cell adhesion_Chemokines and adhesion	1.99E-03
Cell adhesion_Cell-matrix glycoconjugates	4.14E-03
Cell adhesion_ECM remodeling	7.65E-03
Inflammation_Complement system	2.93E-06
Immune response_Th17-derived cytokines	1.25E-05
Proteolysis_ECM remodeling	2.47E-03
Immune response_Phagocytosis	5.17E-03
Proteolysis_Connective tissue degradation	6.38E-03
Inflammation_Innate inflammatory response	2.02E-02
Cell adhesion_Cell-matrix interactions	3.04E-02
Rupture	5.07E-13
Encephalomyelitis, Autoimmune, Experimental	1.71E-12
Nervous System Autoimmune Disease, Experimental	1.71E-12
Cicatrix, Hypertrophic	3.39E-11
Hemolytic-Uremic Syndrome	2.44E-10
Encephalomyelitis	4.54E-10
Polycystic Ovary Syndrome	7.06E-10
Central Nervous System Infections	8.67E-10
Inflammation	8.99E-10

### Comparative Gene Expression Profile of Human TM Cells Phagocytically Challenged Under Physiological and Oxidative Stress Conditions

Oxidative damage is believed to play a major role in the pathogenesis of primary open angle glaucoma (POAG) [18]–[21]. To investigate the potential effect of oxidative stress in phagocytosis, we conducted gene expression profile analysis in confluent cultures of human TM cells grown for two weeks under 40% O_2_ and then challenged for three days either to pHRodo-labeled E. coli or to pigment.

Nine hundred seventy-six and 383 genes were significantly up-regulated and down-regulated, respectively, more than 1.5-fold in TM cells phagocytically challenged to E. coli (Table S6 and Table S7 list genes with fold change >2). Similarly to what we observed under physiological conditions, a lower number of genes were differentially expressed upon phagocytosis of pigment particles (23 genes and 12 genes significantly up-regulated and down-regulated, respectively, more than 1.5 fold, Table S8 and Table S9).

**Figure 5:**
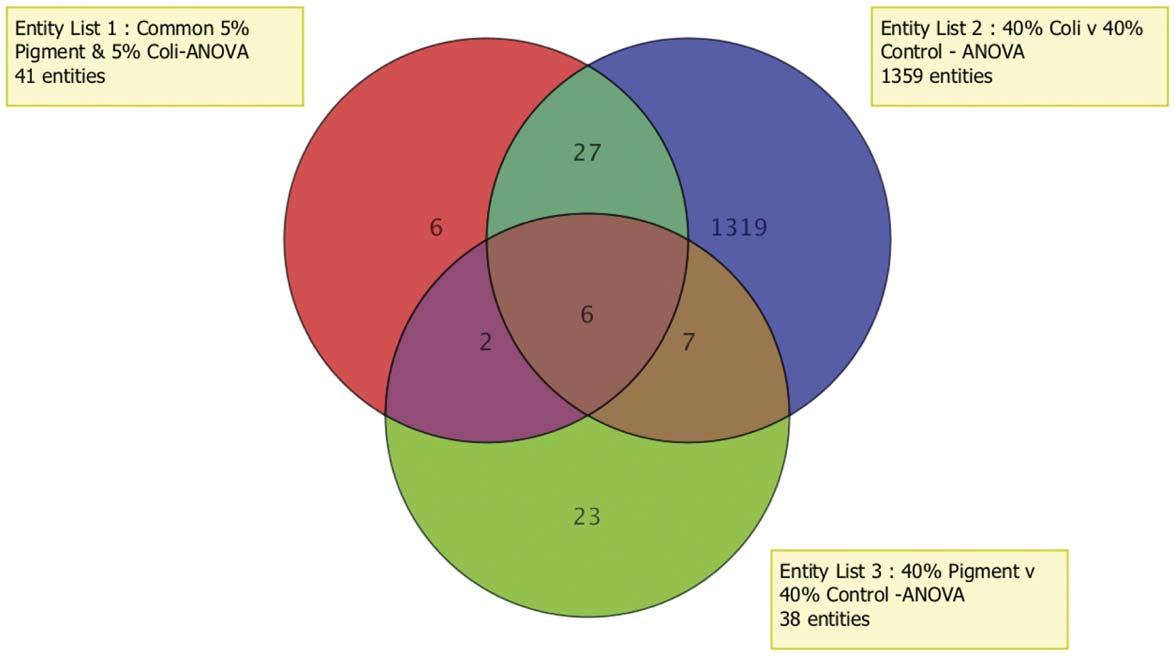
Venn diagram indicating the overlap between genes with differential expression in human TM cells phagocytically challenged with E. coli and pigment under physiological and oxidative stress conditions.

Comparative analysis narrowed to six the number of genes commonly up-regulated or down-regulated (>1.5 fold, p<0.05) upon phagocytosis of E. coli and pigment particles under physiological and oxidative stress conditions (Figure 5, Table 4). Changes in gene expression of MMP1, MMP3, and TNFSF11 were validated by qPCR in both human (Figure 2, stripped bars) and porcine (Figure 3, stripped bars) TM cells. Summary of the expression levels obtained in the qPCR experiments are included as Supporting Information (Table S5).

**Table 4:** Common Genes Differentially Expressed (>1.5 fold, p<0.05) in HTM Cells Phagocytically Challenged Under Physiological and Oxidative Stress Conditions.

Gene Title	Gene Symbol	UniGene ID	Reg	5% O2			40% O2		
				Control	E. Coli	Pigment	Control	E. coli	Pigment
deiodinase, iodothyronine, type II	DIO2	Hs.202354	up	−0.26	1.23	0.30	−1.13	0.70	−0.46
matrix metallopeptidase 1 (interstitial collagenase)	MMP1	Hs.83169	up	−0.65	1.54	0.32	−1.11	0.73	−0.27
matrix metallopeptidase 3 (stromelysin 1, progelatinase)	MMP3	Hs.375129	up	−1.16	2.31	−0.10	−0.76	2.04	0.07
tumor necrosis factor (ligand) superfamily, member 11	TNFSF11	Hs.333791	up	−0.03	1.40	0.86	−1.60	0.02	−0.52
kynureninase (L-kynurenine hydrolase)	KYNU	Hs.470126	up	−1.34	1.52	−0.54	−0.35	2.00	0.28
potassium intermediate/small conductance calcium-activated channel, subfamily N, member 2	KCNN2	Hs.98280	down	0.36	−0.63	−0.20	0.89	−0.18	0.20

### Role of NF-kB in the Transcriptional Activation of MMP1 and MMP3

**Figure 6:**
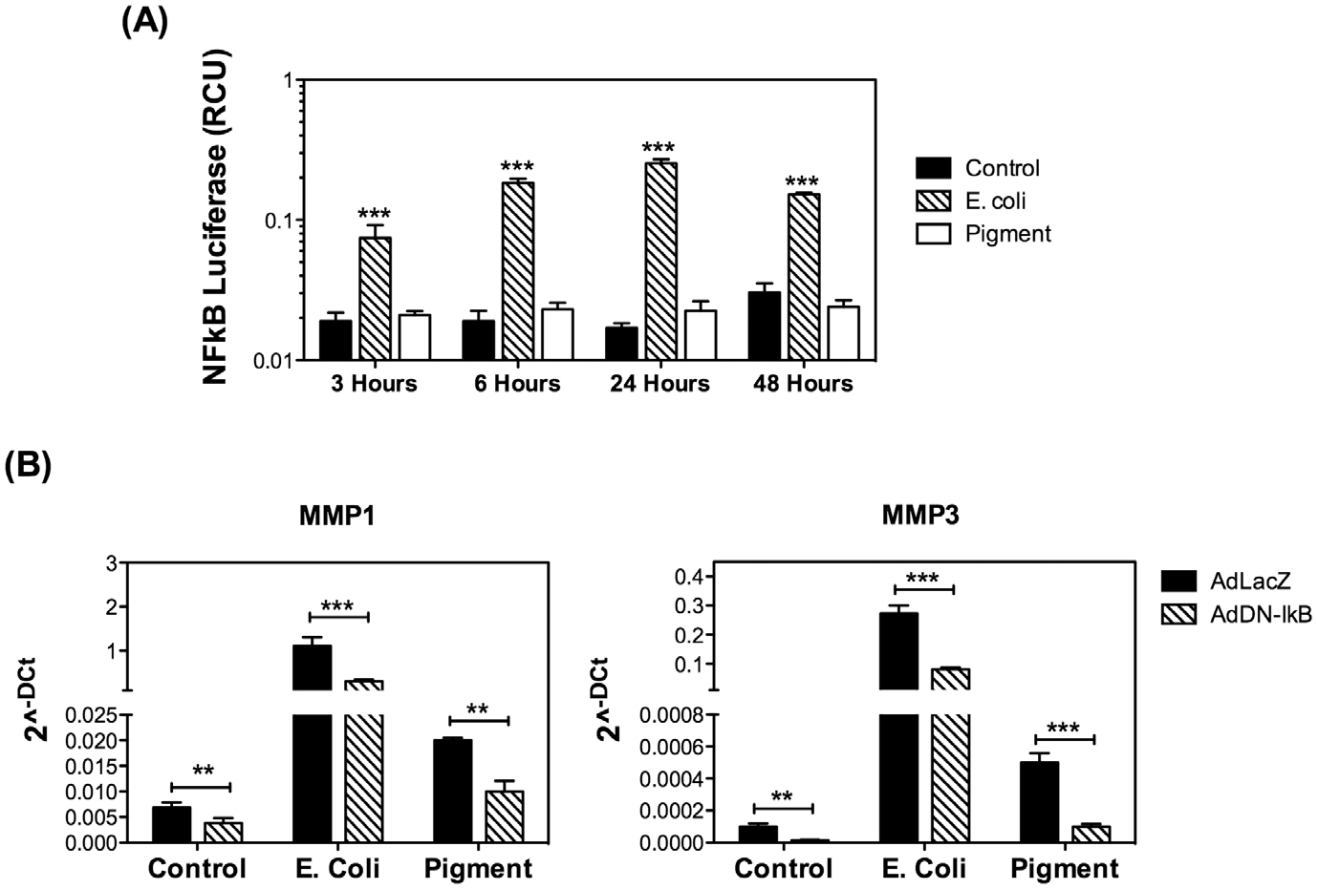
Role of NF-kB in MMP1 and MMP3 upregulation in response to phagocytic challenge. (A) Activation of NF-kB in porcine TM cells phagocytically challenge to E. coli or pigment quantified using a dual luciferase reporter assay. (B) Expression levels of MMP1 and MMP3 mRNAs in porcine TM cells infected with AdLacZ or AdDN-IkB and phagocytically challenged for three days to either E. coli or pigment particles. The expression levels were calculated using the formula 2^−ΔCt^, where Δ_Ct_ = Ct_gene_−Ct _average housekeeping_. β-Actin, GAPDH, and HPRT1 served as internal standard for normalization. Values represent mean ± SD. **p<0.005, ***p<0.0005 (t-test, n = 3).

Metacore analysis revealed NF-kB as the most likely transcription factor regulating the transcriptional activation of several genes whose expression is modified upon phagocytosis, including MMP1 and MMP3. Since chronic activation of NF-kB has been reported in the outflow pathway of glaucoma patients [22], we were particularly interested in confirming this data, obtained by bioinformatics algorithms. For this, we first tested the activation of NF-kB following phagocytic challenge using a luciferase reporter assay. As seen in Figure 6A, phagocytic challenge to E. coli significantly triggered a strong NF-kB activation, which reached a plateau after 24 hours (>10 fold). A more discrete activation of NF-kB, (32.5±5.12% at 24 hours) was observed with engulfment of pigment particles. We additionally evaluated the effects of blocking NF-kB activation on the up-regulation of MMP1 and MMP3 upon phagocytosis. For this, porcine TM cells were infected with a recombinant adenovirus containing either LacZ (AdLAcZ) or the dominant negative mutant of IkB (AdDN-IkB). At two days post-infection (d.p.i.) cells were phagocytically challenged with either E. coli or pigment. Expression levels of MMP1 and MMP3 mRNAs were quantified at day three by qPCR. As shown in Figure 6B, repression of NF-kB translocation significantly inhibited the up-regulation of MMP1 and MMP3 in response to phagocytic challenge.

### Collagenolytic and Caseinolytic Activities in Phagocytically Challenged TM Cells

**Figure 7:**
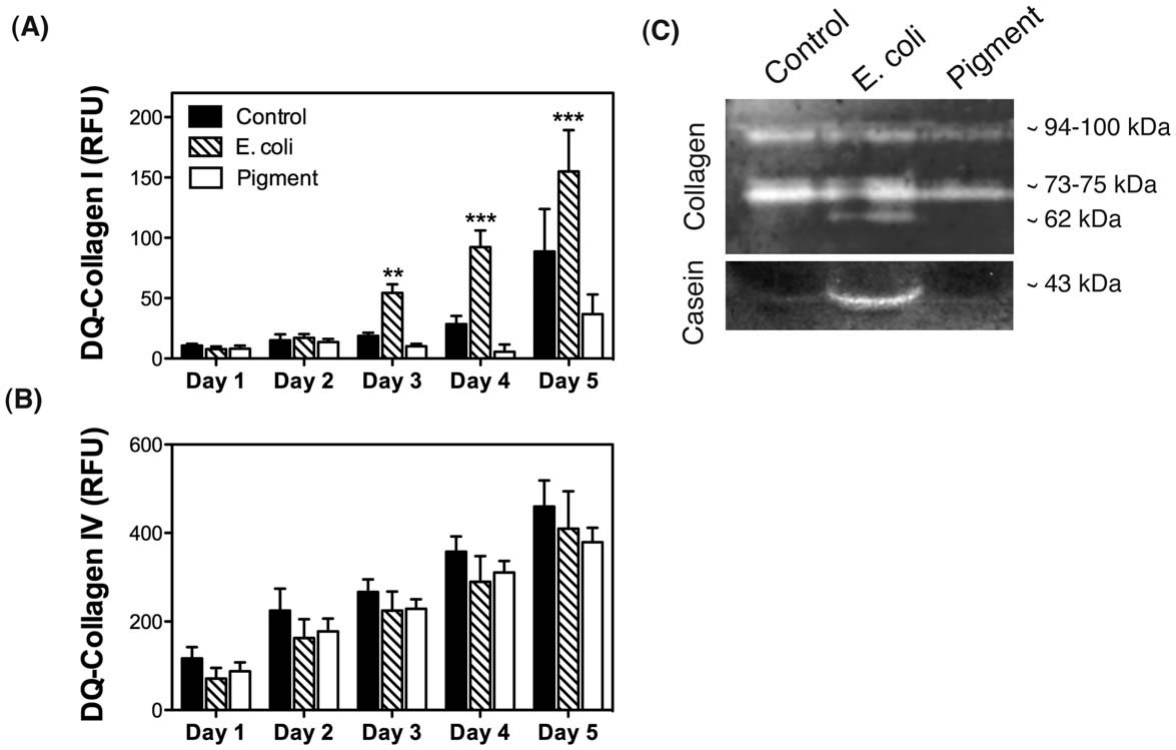
Collagenolytic activity of porcine TM cells phagocytically challenged to E.coli or pigment in the presence of the self-quenched fluorescent substrates DQ-Collagen I (A) or DQ-Collagen IV (B). Values represent mean ± SD. **p<0.005, ***p<0.0005 (t-test, n = 3). (C) Collagenolytic and caseinolytic activities in the culture media of porcine TM cells phagocytically challenged to E.coli or pigment evaluated by in-gel collagen I or casein zymography. Lytic activity is shown as clear bands. Zymograms are representative from three independent experiments.

The proteins coded by MMP1 and MMP3 belong to the matrix metalloproteinases (MMPs) family, a group of zinc-containing endopeptidases that actively participate in the degradation of components of the extracellular matrix (ECM). MMPs have different but overlapping substrate specificity. In particular, MMP1 preferentially cleaves collagen I, whereas MMP3 shows higher specificity for collagen IV [23], [24]. To evaluate whether the observed transcriptional up-regulation of MMP1 and MMP3 might translate into increased collagenolytic activity upon phagocytosis, we monitored the degradation of collagen I and collagen IV in phagocytically challenged porcine TM cells using the quenched fluorescent substrates DQ-collagen I and DQ-collagen IV, respectively. As seen in Figure 7A, phagocytosis of E. coli promoted the degradation of collagen I overtime, demonstrated by the increased in fluorescence resulting from the proteolytic degradation of the substrate. The presence of pigment particles, however, caused a slightly decrease in collagenolytic I activity compared to control. No significant changes in the degradation of collagen IV were observed with any of the phagocytic ligands at the time tested (Figure 7B).

We additionally checked MMPs expression in the culture media by substrate gel zymography (Figure 7C). Collagen I-zymography revealed two major bands of activity at ∼94–100 kDa and ∼73–75 kDa, which correspond to MMP9 and pro-MMP2, respectively. No difference in quantities between the experimental conditions and the control were observed. An additional band at ∼62 kDa was detected in the cultures exposed to E. coli. This proteolytic activity at ∼62 kDa is referred in the literature as active MMP2 [24]. Casein-zymography identified a lysis band of ∼43 kDa in the culture media of cells phagocytically challenged to E. coli, which is consistent with the molecular weight of active MMP1 [25], [26].

## Discussion

Herein, we report for the first time the differential gene expression profile of cultured TM cells phagocytically challenge to E. coli or pigment under physiological and oxidative stress conditions. Our data show the specific transcriptional up-regulation of MMP1 and MMP3, and increased collagenase activity in cultured TM cells following phagocytosis. Moreover, NF-kB was identified as one of the transcription factor mediating such an up-regulation.

The choice of the phagocytic ligands used in this study was based on our own observations (unpublished data) and data from other investigators. Trabecular meshwork cells have been shown to be capable of ingesting a vast variety of materials, including collagen fragments, melanin granules, fibrin, red blood cells, bacteria, zymosan, colloidal carbon, gold particles and latex microspheres [5]–[11]. Because of the immune privilege of the eye, the conventional outflow pathway is not normally exposed to bacterial or fungal infection, unless in some secondary glaucomas (uveitis glaucoma and glaucoma associated with keratitis). However, E. coli constitutes a widely spread and the most commonly used model to study phagocytosis of biotic substrates in all types of phagocytic cells, including TM cells. Although TM cells do not seem to show a marked preference between phagocytic substrates (opsonized versus non-opsonized, biotic versus non-biotic), a different biological response to foreign particles has been noted by a number of investigators. In general, phagocytosis of biotic degradable material, such as zymosan or E.coli particles, elicited an inflammatory response, whereas ingestion of nondegradable material, such as latex beads or pigment, was observed to alter neither trabecular cell function nor morphology [5], [8], [27]. This is in agreement with the data obtained here.

While comparative gene expression profile and functional network analyses demonstrated the activation of pro-inflammatory pathways in TM cells phagocytically challenged to E. coli, we observed a marked reduction in the number of genes differentially expressed upon phagocytosis of pigment particles. The reason for this differential molecular and biological response is currently unknown. It is possible that despite the fact that cultures were challenged with saturated doses of E. coli or pigment particles, they more efficiently phagocytosed E. coli, resulting in higher differences in gene expression. Electron micrographs showed, however, the presence of great amount of engulfed pigment particles within the cells. A more plausible explanation is that the trabecular cell response to foreign particles may vary with the cellular ingestion mechanisms [5] or with the phagocytic receptor. Supporting this, a recent study has shown that the initial receptor-ligand interactions modulate gene expression and phagosomal properties during both early and late stages of phagocytosis [28]. Similarly, studies in insects have also shown that distinct signaling pathways regulate the phagocytic activity of biotic and abiotic components [29], [30].

We identified a number of genes whose expression resulted significantly up-regulated upon phagocytosis of both pigment particles and E. coli. Enrichment pathway analysis clustered those genes into two main categories: immune response and cell adhesion/ECM remodeling. Since oxidative damage has been linked to the pathogenesis of glaucoma [18]–[21], we also evaluated changes in gene expression in oxidatively stressed TM cells with phagocytosis. Cells under oxidative stress demonstrated a slight decrease in the number of genes with differential expression, compared to those under physiological conditions. It is feasible that chronic exposure to oxidative stress might affect the ability of TM cells to engulf foreign material. We did not observe any apparent decrease in the phagocytic capacity of oxidatively stressed TM cells. We believe, however, that the slight reduction in response is associated with the fact that cells under 40% O_2_ displayed per se a pro-inflammatory profile, similar to the one observed upon phagocytic challenge.

A total of five genes, including MMP1, MMP3, TNFSF11, DIO2, and KYNU were identified to be commonly up-regulated after phagocytosis of E. coli and pigment particles under physiological and oxidative stress conditions. Just one gene, KCNN2, was found down-regulated in all different conditions. Up-regulation of MMP1, MMP3, and TNSF11 with phagocytosis was also confirmed in cultured porcine TM cells. The concordance between the data on the two species might imply that the differences in the post-mortem times at which porcine eyes and human eyes are procured cause no major changes in the biological response of TM cells.

The potential roles of DIO2, KYNU, and KCNN2 in phagocytosis and/or outflow tissue physiology are not clear. KCNN2 codes an integral membrane protein that forms a voltage-independent calcium-activated channel. Activation of large-conductance calcium- and voltage-activated potassium channels (BKCa) have been shown to increase outflow facility and decrease cell volume, suggesting that K(+) efflux regulate TM cell function [31]. The protein encoded by DIO2 gene belongs to the iodothyronine deiodinase family, which is involved in the activation of thyroid hormone. Genetic association studies have identified DIO2 as an osteoarthritis susceptibility gene. It has been recently shown that inflammatory signals as well as bacterial lipopolysaccharide up-regulate DIO2 expression via NF-κB [32], [33]. Kynureninase is an enzyme involved in the biosynthesis of NAD cofactors from tryptophan through the kynurenine pathway. Although chronic up-regulation of KYNU following phagocytosis has been also recently reported in brain microglial cells challenged to alpha beta amyloid [34], its role in phagocytosis has not been defined. Interestingly, the latter study showed increased expression of MMP1 and MMP3 mRNAs upon phagocytosis of alpha beta amyloid [34]. Likewise, up-regulated MMP3 expression and activity has been described upon phagocytosis of apoptotic cholangiocytes by macrophages [35]. MMP1 and MMP3 are members of the matrix metalloproteinases family, a class of proteases that participate in the hydrolysis of components of the ECM. MMP1, also known as interstitial collagenase, is able to cleave interstitial collagens I, II, and III, as well as digest certain other ECM and non-ECM proteins. MMP3 (or stromelysin 1) breaks down ECM components such as collagen IV, laminin, and fibronectin [23], [24], [36]. Collagens, in particular collagen I and collagen IV, are the major components of the trabecular beams and basal laminal [37].

Quantification of the collagenolytic activity using DQ-Collagen I and DQ-collagen IV showed increased degradation of collagen I, but not collagen IV, in porcine TM cells phagocytically challenged to E.coli, which might presumably result from increased MMP1 activity. Despite the observed higher MMP1 and MMP3 mRNA levels, increased collagenase activity was not detected with phagocytosis of pigment particles. Similar results were obtained by substrate gel zymography. Culture media from porcine TM cells challenged to E.coli showed caseinolytic and collagenolytic activities at ∼43 kDa (MMP1) and ∼62 kDa (active MMP2), respectively. However, these were not observed in the culture media from control cells or in the cells challenged to pigment. These findings are not entirely surprising since the activity of MMPs is tightly regulated at three different levels: transcriptional regulation, pro-enzyme activation, and inhibition of proteolytic activity by endogenous inhibitors [23], [36]. Additional experiments aimed at analyzing in more detail the regulation of MMPs with phagocytosis, as well as the contribution of MMP1 and MMP3 in the observed collagenolytic and caseolytic activities are required before reaching further conclusions. Very interestingly, decreased levels of fibronectin and laminin, as well as increased MMP2 activity has been also reported in bovine TM cells phagocytically challenged to latex microspheres [16].

Tumor necrosis factor ligand superfamily member 11 (TNFSF11) was also found to be consistenly up-regulated with E. coli and pigment under physiological and oxidative stress conditions. TNFSF11, known as receptor activator of nuclear factor kappa-B ligand (RANKL), is a member of the tumor necrosis factor cytokine family, which is tightly involved in mediating the activation of NF-kB [38]. Remarkably, metacore pathway analysis identified NF-kB as the most likely transcription factor regulating the transcriptional activation of several of the genes with altered expression following phagocytic challenge, including MMP1 and MMP3. A strong activation of NF-kB was observed in porcine TM cells upon phagocytosis of E. coli. Phagocytosis of pigment particles elicited a much lesser activation of NF-kB, which might explain the lower up-regulation of MMP1 and MMP3 with phagocytosis of pigment compared to E. coli. In both cases, blockage of NF-kB activation using AdDN-IkB significantly decreased the induction of MMP1 and MMP3 with phagocytic challenge. Whether TNFSF11 is involved in the up-regulation of MMP1 and MMP3 via NF-κB needs yet to be established.

Interestingly, chronic activation of NF-kB and up-regulated MMP1 expression have been reported in the glaucomatous TM tissue [22], [39]. Moreover, immunohistochemical analysis showed the protein levels of both, MMP1 and MMP3, to be increased in the outflow pathway of POAG patients compared to controls. Strikingly, the staining intensity of MMP1 and MMP3 in the outflow pathway of exfoliating glaucoma, a type of secondary glaucoma characterized by the extracellular accumulation of non-internalized exfoliating material in the angle chamber, was significantly diminished compared to POAG [40].

In summary, our data demonstrate the up-regulation of several genes able to modify the ECM content in cultured human and porcine TM cells following phagocytic challenge. On the one hand, such an up-regulation of ECM remodeling genes might explain the detachment and loss of cell-matrix cohesiveness reported in TM cells with phagocytosis *in vitro* and *in vivo* [5], [7]–[9], [15]–[17]. This loss of cells with phagocytosis may be a factor contributing to the decreased in cellularity observed in the TM in glaucoma [41], [42], and have a detrimental impact on the ability of the TM to function as a biological filter. On the other hand, because of the relevance of the ECM to the regulation of outflow facility [43], and the key role of MMPs in the turnover and maintenance of the trabecular meshwork's ECM [44], [45], our results suggest a novel role of phagocytosis in the outflow pathway tissue physiology. Futures studies will be directed at confirming the expression data obtained in this study using cultured TM cells in *in vivo* conditions. Also, based on the differential biological response elicited with phagocytic challenge to E. coli versus pigment particles, caution should be taken when analyzing and comparing experimental data obtained using different phagocytic ligands.

## Methods

### Cell Culture

Primary cultures of porcine and human TM cells were prepared and maintained as previously described [46]. Briefly, the TM was dissected and digested with 2 mg/mL of collagenase for 1 hour at 37°C. The digested tissue was placed in gelatin-coated 35 mm dishes and cultivated in low glucose Dulbecco's Modified Eagle Medium (DMEM) with L-glutamine and 110 mg/L sodium pyruvate, supplemented with 10% fetal bovine serum (FBS), 100 mM non-essential amino acids, 100 units/ml penicillin, 100 mg/ml streptomycin sulfate and 0.25 mg/ml amphotericin B; all the reagents were obtained from Invitrogen (Carlsbad, CA). Cells were maintained and propagated until passage three at 37°C in a humidified air with 5% CO_2_ incubator. Cell lines were subcultivated 1∶2 when confluent. Primary cultures of porcine TM cells were prepared from porcine cadaver eyes obtained from a local abattoir (City Packing CO, Burlington, NC) less than five hours post-mortem. Primary cultures of human TM cells were prepared from cadaver eyes (ages 30–60, no history of eye disease) obtained less than 48 hours post-mortem from the North Carolina Eye Bank (NCEB) and National Disease Research Interchange (NDRI). The protocols involving the use of human tissue were consistent with the tenets of the Declaration of Helsinki. At least, three independent batches of TM cells were used in these experiments. All the cell lines were tested positive for matrix gla protein, chitinase 3 like-1, and increased MYOC expression in response to corticoids. Chronic oxidative stress was induced by subjecting TM cells to normobaric hyperoxia conditions. For this, confluent cultures of TM cells at passage four were grown for two weeks at 40% O_2_ and 5% CO_2_. Control cultures were grown under physiological oxygen conditions (5% O_2_, 5% CO_2_) in a triple gas incubator [46].

### Pigment Isolation

Pigment was harvested from porcine eyes as follows [27]. Eyes were cleaned and sterilized. The iris and the ciliary body were dissected and placed in 10 mL sterile water. Tissues were vortexed and smashed to release the pigment, and then centrifuged at 120×g for five minutes to eliminate cellular debris. The supernatant containing the pigment was collected and spun at 840×g for 15 minutes. The pigment pellet was resuspended in sterile PBS and stored at −80°C. The concentration of pigment particles was calculated using a hematocytometer. The pigment fraction obtained using this technique was pure and no cells or tissues debris could be observed under the microscope. Only one batch of pigment was required to perform all the experiments.

### Phagocytic Challenge

Porcine or human TM cells were phagocytically challenged with either pHRodo-labeled E. coli bioparticles (1×10 ^6^ particles/mL, Invitrogen, Carlsbad, CA) or porcine pigment (1×10 ^6^ particles/mL) isolated as described earlier.

### Electron Microscopy

Cells were washed twice in PBS and fixed in 2.5% glutaraldehyde in 0.1 M cacodylate buffer (pH 7.2). Fixed cells were then detached by gentle scraping, pelleted, post-fixed in 1% osmium tetroxide in 0.1 M cacodylate buffer, and processed for transmission electron microscopy in the Morphology Facility at Duke Eye center. Thin sections (65 nm) were examined in a JEM-1200EX electron microscopy.

### RNA Isolation

Following the experimental conditions, TM primary cultures were extensively washed with cold PBS and fixed in RNAlater (Qiagen). Total RNA was isolated using RNeasy kit (Qiagen, Valencia, CA), following the manufacturer's protocol, and then treated with DNase I. RNA yields were determined using the RiboGreen fluorescent dye (Molecular Probes, Eugene, OR). RNA quality was confirmed using the Agilent 2100 Bioanalyzer.

### Microarray Analysis

Total RNA (10 µg) from human TM primary cultures phagocytically challenged for two days to either E. coli or pigment under physiological or oxidative stress conditions were independently hybridized to Affymetrix Human Genome U133 Plus 2.0 microarrays following the manufacturer's instructions. Data analysis was performed using the GeneSpring Software 10.0 (Silicon Genetics, Redwood City, CA). Raw data from the twelve hybridizations were normalized to the 50th percentile per chip and to the median per gene. Normalized mean values for the six individual experimental groups (5%O_2_-Control, 5%O_2_-E.coli, 5%O_2_-Pigment, 40%O_2_-Control, 40%O_2_-E.coli, 40%O_2_-Pigment) were generated for the experimental interpretation. The analysis was performed using the Replicates interpretation. Genes with a differential gene expression were selected and then filtered on flags to retain the genes that were presented in at least one of the conditions. Since some genes were represented in the arrays in more than one spot, we verified a consistent differential expression in all the spots to eliminate false positives. The statistical significance of the differences was evaluated by using the cross-gene-error model in combination with one-way ANOVA (p<0.05) and Bonferroni multiple testing correction. Since some genes are represented in the arrays in more than one spot, we verified a consistent differential expression in all the spots to eliminate false positives. Microarray data were deposited in the GEO database (#GSE32169) and followed MIAME requirements.

### Integrated Pathway Enrichment Analysis

Integrated pathway enrichment analysis of the set of genes differentially expressed upon phagocytosis was performed by using the knowledge-based canonical pathways and endogenous metabolic pathways in GeneGo MetaCore (http://www.genego.com/metacore.php). For network analysis, transcription regulation, and direct interactions the workflow tool was used.

### Quantitative PCR

First-strand cDNA was synthesized from total RNA (1 µg) by reverse transcription using oligo(dT) primer and Superscript II reverse transcriptase (Invitrogen, Carlsbad, CA). Real-time PCRs were performed in a 20 µL mixture containing 1 µL of the cDNA preparation diluted five times, 10 µL iQ SYBR Green Supermix (Bio-Rad, Hercules, CA), and 500 nm of each primer, in the BIO-RAD iCycler iQ system (Bio-Rad, Hercules, CA) using the following PCR parameters: 95°C for 5 min, followed by 50 cycles of 95°C for 15 s, 60°C for 15 s, and 72°C for 15 s. The fluorescence threshold value (Ct) was calculated using the iCycle iQ system software. The absence of nonspecific products was confirmed by both the analysis of the melt curves and by electrophoresis in 3% Super AcrylAgarose gels. The average Ct value of the following housekeeping genes (β-Actin, GAPDH, and HPRT1) served as internal standard of mRNA expression. The expression levels were calculated using the formula 2^−ΔCt^, where ΔCt = Ct_gene_−Ct _average housekeeping_. The sequences of the primers used for the amplifications are shown in Table 5.

**Table 5:** Primer Sequences.

		Forward	Reverse
**Human**	**MMP1**	CCAGGCCCAGGTATTGGAGGGG	GGCCGAGTTCATGAGCCGCA
	**TFPI2**	GCTGTGGAGGGAATGACAAT	TCCGGATTCTACTGGCAAAG
	**LAMC2**	CGCAGCTCTGCAGAATACAG	AGACCCATTTCGTTGGACAG
	**TNFSF11**	CGGGGTGACCTTATGAGAAA	GCGCTAGATGACACCCTCTC
	**EDN3**	AGGCTGCATGGTGTATGTCA	TCTGCCAAAATCCCATAAGC
	**GAPDH**	ACAGTCAGCCGCATCTTCTT	ACGACCAAATCCGTTGACTC
**Porcine**	**MMP1**	CCAGGCCCAGGTATTGGAGGGG	GGCCCAGTTCATGAGCAGCCA
	**TNFSF11**	GCCCTTTGCCCACCTCACGA	TCTTGGCCCAACCTCGGTCA
	**GAPDH**	TGTCCCCACCCCCAACGTGT	CCCTCGGACGCCTGCTTCAC
**Human and Porcine**	**ACT**	TCCCTGGAGAAGAGCTACGA	AGGAAGGAAGGCTGGAAGAG
	**HPRT1**	ACACTGGCAAAACAATGCAA	ACACTTCGAGGGGTCCTTTT
	**MMP3**	GGAGGTGACGGGGAAGCTGG	GCCAGGAAAGGTGCTGAAGT

### NF-κB Luciferase Reporter Assay

Activation of NF-κB was monitored using a luciferase reporter assay (Dual-Luciferase Reporter Assay System, Promega). For this, porcine TM cells were transiently transfected with 1 µg of NF-κB firefly luciferase and 1 µg of renilla luciferase reporter plasmids, using the Amaxa Nucleoporation System (Basic Nucleofector Kit, T23 program). Two days after transfection, cells were phagocytically challenged to E. coli or pigment. Luciferase activity in cell lysates harvested at the indicated times was quantified following the manufacturer's instructions.

### Adenoviral Infection of Trabecular Meshwork Cells

Porcine TM cells at passage 4 were infected with replication deficient adenoviruses encoding either LacZ or mutant IκB (m.o.i = 5 pfu/cell) [47]. Briefly, viral suspensions diluted in a small volume of serum-free media were allowed to adsorb to the cell surface membrane by incubation during 90 minutes at 37°C, 5% CO^2^ with shaking every 15 minutes to get a homogeneous distribution of the viral particles in the plate. After the adsorption period, regular culture medium was added to plates.

### Substrate Gel Zymography

Collagenolytic and caseinolytic activities in the culture media were evaluated by collagen I gel zymography and casein gel zymography, respectively. Supernatant samples (25 µl) were mixed with equal volumes of 2× zymography sample buffer (125 mM Tris-HCl, pH 6.8, 50% glycerol, 8% SDS, 0.02% bromophenol blue), loaded onto SDS-PAGE gels containing casein or collagen I under nonreducing conditions, and electrophoresed with 2.5 mM Tris-HCl, 19.2 mM glycine, 0.01% SDS, pH 8.3, at 100 V. After electrophoresis, gels were washed with 1× renaturing buffer and 1× development buffer for 30 minutes each, and incubated overnight in zymogram development buffer (Bio-Rad). Gels were then stained with Coomassie blue R-250 followed by destaining with 55% methanol and 7% acetic acid. Areas of MMP activity appeared as clear bands. Pre-formulated zymography buffers and pre-cast casein gels were purchased from Bio-Rad (Hercules, CA). Collagen I-containing gels were prepared in the laboratory by adding 1 mL of 0.1% Collagen solution, type 1 from calf skin (sigma cat.# C8919-20ML) in 10 mL of 10% acrylamide gel [48].

### Collagenase Activity

Collagenase activity was monitored using DQ collagen, type I and type IV, fluorescein conjugates (Invitrogen, Carlsbad, CA) as follows. Confluent cultures of porcine TM cells grown in 96-well plate were phagocytically challenged to either E. coli or pigment in the presence of vehicle, DQ-Collagen I (10 µg/mL), or DQ-Collagen IV (10 µg/mL). Fluorescence peptides released by the enzymatic cleavage of the substrates were measured in a microplate reader at the indicated times (Em: 495 nm; Exc: 515 nm). All values were corrected for background fluorescence.

### Statistic Analysis

All experimental procedures were repeated at least three times in independent experiments using different cell lines. The percentage of increase of the experimental conditions compared to the control was calculated and averaged. Data are represented as mean ± SD. Statistical significance was calculated using Student's t-test for two groups comparisons using the software GraphPad Prism. A probability less than 5% was considered statistically significant.

## Supporting Information

Figure S1 **Expression Levels of MMP1, MMP3, and TNFSF11 in Phagocytically Challenged porcine TM cells:** Confluent cultures of porcine TM cells grown for two weeks under physiological (black bars) and oxidative stress conditions (stripped bars) were phagocytically challenged to E. coli or pigment particles. mRNA levels of MMP1, MMP3, and TNFSF11 were quantified by real-time PCR at day 1, 2, and 3 post-challenge. The expression levels were calculated using the formula 2^−ΔCt^, where ΔCt = Ct_gene_−Ct _average housekeeping_. β-Actin, GAPDH, and HPRT1 served as internal standard for normalization. Values represent mean ± SD. * p<0.05, ** p<0.005, *** p<0.0005 (t-test, n = 3).
(TIFF)

Table S1 **List of the genes significantly upregulated (>2 fold, p<0.05) in HTM Cells phagocytically challenged to E. coli under physiological conditions.** Confluent cultures of HTM cells were grown for two weeks under physiological 5% O_2_ atmosphere, and then phagocytically challenged to E. coli bioparticles. Changes in gene expression at day 3 post-phagocytic challenge were evaluated by gene array using Affymetrix Human Genome U133 Plus 2.0 chips, and analyzed by Genespring Software.
(PDF)

Table S2 **List of the genes significantly downregulated (>2 fold, p<0.05) in HTM Cells phagocytically challenged to E. coli under physiological conditions.** Confluent cultures of HTM cells were grown for two weeks under physiological 5% O_2_ atmosphere, and then phagocytically challenged to E. coli bioparticles. Changes in gene expression at day 3 post-phagocytic challenge were evaluated by gene array using Affymetrix Human Genome U133 Plus 2.0 chips, and analyzed by Genespring Software.
(PDF)

Table S3 **List of genes significantly upregulated (>1.5 fold, p<0.05) in HTM Cells phagocytically challenged to pigment particles under physiological conditions.** Confluent cultures of HTM cells were grown for two weeks under physiological 5% O_2_ atmosphere, and then phagocytically challenged to pigment particles. Changes in gene expression at day 3 post-phagocytic challenge were evaluated by gene array using Affymetrix Human Genome U133 Plus 2.0 chips, and analyzed by Genespring Software.
(PDF)

Table S4 **List of genes significantly downregulated (>1.5 fold, p<0.05) in HTM Cells phagocytically challenged to pigment particles under physiological conditions.** Confluent cultures of HTM cells were grown for two weeks under physiological 5% O_2_ atmosphere, and then phagocytically challenged to pigment particles. Changes in gene expression at day 3 post-phagocytic challenge were evaluated by gene array using Affymetrix Human Genome U133 Plus 2.0 chips, and analyzed by Genespring Software.
(PDF)

Table S5 **Quantitative real-time PCR confirmation of selected genes with differential expression in phagocytically challenged human and porcine TM cells under physiological and oxidative stress conditions.** The expression levels were calculated using the formula 2-ΔCt, where ΔCt = Ctgene-Ct average housekeeping. β-Actin, GAPDH, and HPRT1 served as internal standard for normalization. Values represent mean ± SD, t-test, n = 3. (*) compares phagocytically challenged versus control cultures; (#) compares oxidatively stressed versus cultures grown under physiological conditions.
(PDF)

Table S6 **List of the genes significantly upregulated (>2 fold, p<0.05) in HTM Cells phagocytically challenged to E. coli under oxidative stress conditions.** Confluent cultures of HTM cells were grown for two weeks under oxidative 40% O_2_ atmosphere, and then phagocytically challenged to E. coli bioparticles. Changes in gene expression at day 3 post-phagocytic challenge were evaluated by gene array using Affymetrix Human Genome U133 Plus 2.0 chips, and analyzed by Genespring Software.
(PDF)

Table S7 **List of the genes significantly downregulated (>2 fold, p<0.05) in HTM Cells phagocytically challenged to E. coli under oxidative stress conditions.** Confluent cultures of HTM cells were grown for two weeks under oxidative 40% O_2_ atmosphere, and then phagocytically challenged to E. coli bioparticles. Changes in gene expression at day 3 post-phagocytic challenge were evaluated by gene array using Affymetrix Human Genome U133 Plus 2.0 chips, and analyzed by Genespring Software.
(PDF)

Table S8 **List of the genes significantly upregulated (>2 fold, p<0.05) in HTM Cells phagocytically challenged to pigment under oxidative stress conditions.** Confluent cultures of HTM cells were grown for two weeks under oxidative 40% O_2_ atmosphere, and then phagocytically challenged to pigment particles. Changes in gene expression at day 3 post-phagocytic challenge were evaluated by gene array using Affymetrix Human Genome U133 Plus 2.0 chips, and analyzed by Genespring Software.
(PDF)

Table S9 **List of the genes significantly downregulated (>2 fold, p<0.05) in HTM Cells phagocytically challenged to pigment under oxidative stress conditions.** Confluent cultures of HTM cells were grown for two weeks under oxidative 40% O_2_ atmosphere, and then phagocytically challenged to pigment particles. Changes in gene expression at day 3 post-phagocytic challenge were evaluated by gene array using Affymetrix Human Genome U133 Plus 2.0 chips, and analyzed by Genespring Software.
(PDF)
