# Supplementary figures and images for: Up-Regulated Expression of Extracellular Matrix Remodeling Genes in Phagocytically Challenged Trabecular Meshwork Cells
Source: PLoS One. 2012 Apr 18;7(4):e34792. doi: 10.1371/journal.pone.0034792 (PMC3329506; doi:10.1371/journal.pone.0034792)

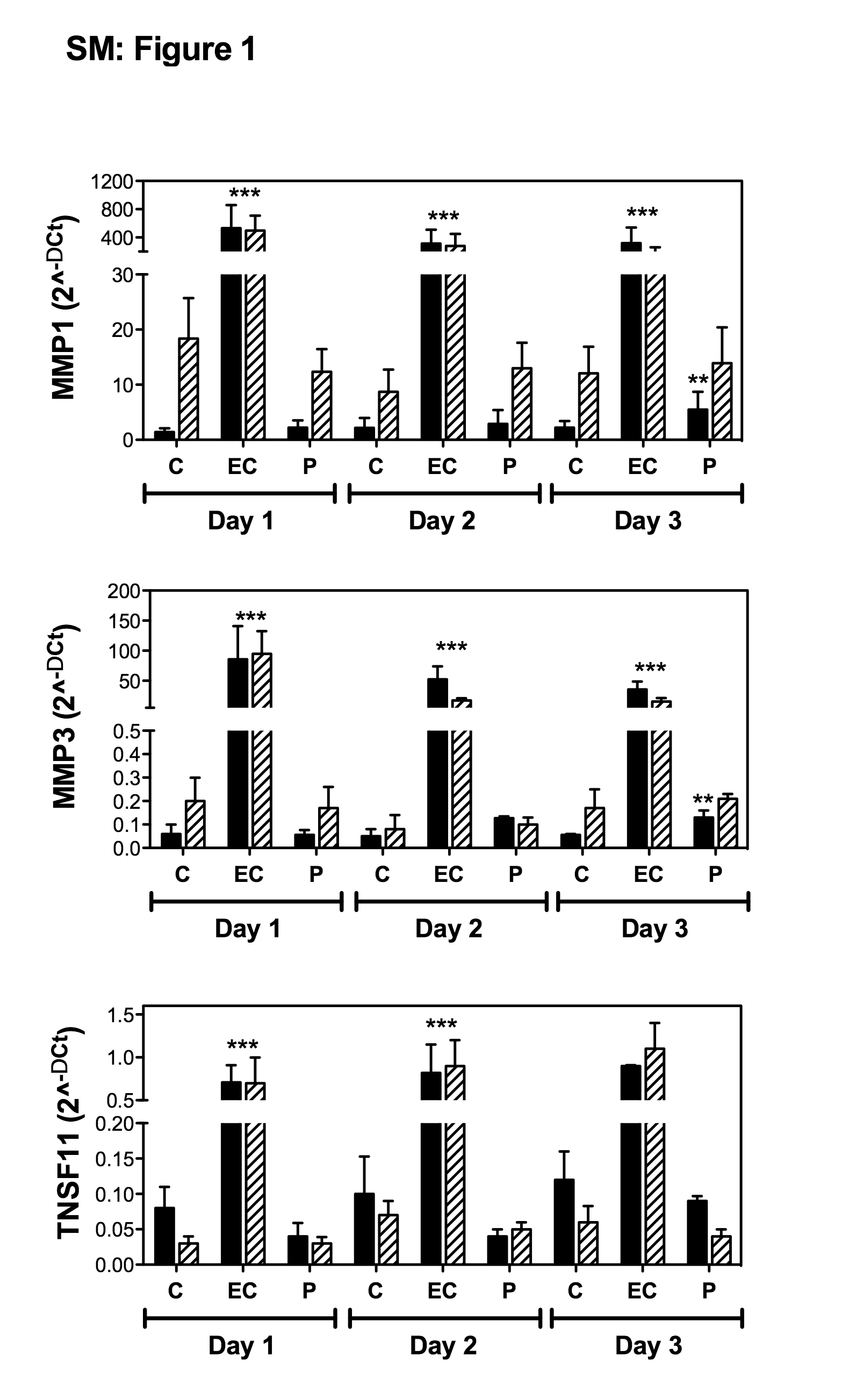

Supplement: Figure S1 — Expression Levels of MMP1, MMP3, and TNFSF11 in Phagocytically Challenged porcine TM cells: Confluent cultures of porcine TM cells grown for two weeks under physiological (black bars) and oxidative stress conditions (stripped bars) were phagocytically challenged to E. coli or pigment particles. mRNA levels of MMP1, MMP3, and TNFSF11 were quantified by real-time PCR at day 1, 2, and 3 post-challenge. The expression levels were calculated using the formula 2−ΔCt, where ΔCt = Ctgene−Ct average housekeeping. β-Actin, GAPDH, and HPRT1 served as internal standard for normalization. Values represent mean ± SD. * p<0.05, ** p<0.005, *** p<0.0005 (t-test, n = 3). (TIFF) [file pone.0034792.s001.tif]
