# Supplementary material for: Up-Regulated Expression of Extracellular Matrix Remodeling Genes in Phagocytically Challenged Trabecular Meshwork Cells
Source: PLoS One. 2012 Apr 18;7(4):e34792. doi: 10.1371/journal.pone.0034792 (PMC3329506; doi:10.1371/journal.pone.0034792)
Supplement: Table S1 — List of the genes significantly upregulated (>2 fold, p<0.05) in HTM Cells phagocytically challenged to E. coli under physiological conditions. Confluent cultures of HTM cells were grown for two weeks under physiological 5% O2 atmosphere, and then phagocytically challenged to E. coli bioparticles. Changes in gene expression at day 3 post-phagocytic challenge were evaluated by gene array using Affymetrix Human Genome U133 Plus 2.0 chips, and analyzed by Genespring Software. (PDF) [file pone.0034792.s002.pdf]

SM-Table 1: Genes Significantly Upregulated (>2 fold, $p<0.05$) in HTM Cells Phagocytically Challenged to E. coli Under Physiological Conditions

Gene Title	Gene Symbol	UniGene ID	Fold	PValue	Chromosomal Location
chemokine (C-X-C motif) ligand 11	CXCL11	Hs.632592	177.85	1.8E-03	chr4q21.2
interferon, alpha-inducible protein 27	IFI27	Hs.532634	75.68	2.1E-03	chr14q32
myxovirus (influenza virus) resistance 1, interferon-inducible protein p78 (mouse)	MX1	Hs.517307	57.25	2.1E-03	chr21q22.3
chemokine (C-X-C motif) ligand 5	CXCL5	Hs.89714	54.52	2.5E-03	chr4q12-q13
chemokine (C-C motif) ligand 20	CCL20	Hs.75498	48.05	3.5E-03	chr2q33-q37
interferon-induced protein 44-like	IFI44L	Hs.389724	47.48	1.8E-03	chr1p31.1
bone marrow stromal cell antigen 2	BST2	Hs.118110	43.91	1.9E-03	chr19p13.2
chemokine (C-X-C motif) ligand 2	CXCL2	Hs.75765	43.51	1.9E-03	chr4q21
chemokine (C-C motif) ligand 8	CCL8	Hs.271387	39.29	3.4E-03	chr17q11.2
chemokine (C-X-C motif) ligand 3	CXCL3	Hs.89690	37.79	2.7E-03	chr4q21
radical S-adenosyl methionine domain containing 2	RSAD2	Hs.17518	36.87	3.5E-03	chr2p25.2
2',5'-oligoadenylate synthetase 1, 40/46kDa	OAS1	Hs.524760	34.84	4.1E-03	chr12q24.1
ISG15 ubiquitin-like modifier	ISG15	Hs.458485	33.80	2.1E-03	chr1p36.33
2'-5'-oligoadenylate synthetase 2, 69/71kDa	OAS2	Hs.414332	28.77	1.8E-03	chr12q24.2
hect domain and RLD 6	HERC6	Hs.529317	25.85	2.3E-03	chr4q22.1
chemokine (C-C motif) ligand 7	CCL7	Hs.251526	24.14	1.8E-03	chr17q11.2-q12
superoxide dismutase 2, mitochondrial	SOD2	Hs.487046	24.05	3.6E-03	chr6q25.3
interleukin 33	IL33	Hs.348390	22.50	2.2E-03	chr9p24.1
chemokine (C-X-C motif) ligand 10	CXCL10	Hs.632586	21.74	2.1E-03	chr4q21
interleukin 8	IL8	Hs.624	20.68	5.8E-03	chr4q13-q21
cytidine monophosphate (UMP-CMP) kinase 2, mitochondrial	CMPK2	Hs.7155	19.93	4.0E-03	chr2p25.2
Ets homologous factor	EHF	Hs.653859	19.32	1.2E-03	chr11p12
interleukin 6 (interferon, beta 2)	IL6	Hs.654458	18.83	2.7E-03	chr7p21
chromosome 15 open reading frame 48	C15orf48	Hs.112242	18.02	3.2E-03	chr15q21.1
chemokine (C-X-C motif) ligand 1 (melanoma growth stimulating activity, alpha)	CXCL1	Hs.789	16.33	3.9E-03	chr4q21
myxovirus (influenza virus) resistance 2 (mouse)	MX2	Hs.926	15.86	2.9E-03	chr21q22.3
complement component 2 /// complement factor B	C2 /// CFB	Hs.408903	15.74	2.1E-03	chr6p21.3
interferon-induced protein with tetratricopeptide repeats 1	IFIT1	Hs.20315	14.39	2.7E-03	chr10q25-q26
interferon, alpha-inducible protein 6	IFI6	Hs.523847	14.31	2.1E-03	chr1p35
interferon-induced protein with tetratricopeptide repeats 3	IFIT3	Hs.714337	14.04	2.1E-03	chr10q24
solute carrier family 39 (zinc transporter), member 8	SLC39A8	Hs.288034	13.42	4.7E-03	chr4q22-q24
matrix metallopeptidase 12 (macrophage elastase)	MMP12	Hs.1695	13.16	2.3E-03	chr11q22.3
metallothionein 1M	MT1M	Hs.647370	13.08	6.0E-03	chr16q13

SM-Table 1: Genes Significantly Upregulated (>2 fold, $p<0.05$) in HTM Cells Phagocytically Challenged to E. coli Under Physiological Conditions

Gene Title	Gene Symbol	UniGene ID	Fold	PValue	Chromosomal Location
stanniocalcin 1	STC1	Hs.25590	12.64	1.9E-03	chr8p21-p11.2
interferon induced with helicase C domain 1	IFIH1	Hs.163173	12.29	2.1E-03	chr2q24
tumor necrosis factor, alpha-induced protein 3	TNFAIP3	Hs.211600	11.98	2.3E-03	chr6q23
230746_s_at		Hs.713064	11.70	1.8E-03	
chemokine (C-C motif) ligand 2	CCL2	Hs.303649	11.65	3.7E-03	chr17q11.2-q12
matrix metallopeptidase 3 (stromelysin 1, progelatinase)	MMP3	Hs.375129	11.20	3.8E-03	chr11q22.3
hypothetical protein LOC387763	LOC387763	Hs.714890	11.13	4.2E-03	chr11p11.2
hydroxysteroid (11-beta) dehydrogenase 1	HSD11B1	Hs.195040	10.86	1.7E-03	chr1q32-q41
SAM domain and HD domain 1	SAMHD1	Hs.580681	10.80	3.5E-03	chr20pter-q12
complement component 3	C3	Hs.529053	10.59	3.4E-03	chr19p13.3-p13.2
nuclear factor of kappa light polypeptide gene enhancer in B-cells inhibitor, zeta	NFKBIZ	Hs.319171	10.27	4.2E-03	chr3p12-q12
cathepsin S	CTSS	Hs.181301	10.10	3.6E-03	chr1q21
tumor necrosis factor, alpha-induced protein 6	TNFAIP6	Hs.437322	10.06	1.8E-03	chr2q23.3
chemokine (C-C motif) ligand 5	CCL5	Hs.514821	9.97	3.4E-03	chr17q11.2-q12
tryptophan 2,3-dioxygenase	TDO2	Hs.183671	9.87	4.6E-03	chr4q31-q32
baculoviral IAP repeat-containing 3	BIRC3	Hs.127799	9.07	1.2E-03	chr11q22
tissue factor pathway inhibitor 2	TFPI2	Hs.438231	8.97	6.7E-03	chr7q22
234987_at		Hs.660221	8.56	1.9E-03	
mitogen-activated protein kinase kinase kinase 8	MAP3K8	Hs.432453	8.50	6.5E-03	chr10p11.23
chromosome 8 open reading frame 4	C8orf4	Hs.591849	8.19	1.2E-03	chr8p11.2
2'-5'-oligoadenylate synthetase 3, 100kDa	OAS3	Hs.528634	8.19	1.1E-03	chr12q24.2
serpin peptidase inhibitor, clade B (ovalbumin), member 2	SERPINB2	Hs.594481	8.10	3.5E-03	chr18q21.3
mesenchyme homeobox 1	MEOX1	Hs.438	7.84	1.2E-03	chr17q21
myocardin	MYOCD	Hs.567641	7.82	4.0E-03	chr17p11.2
interleukin 11	IL11	Hs.467304	7.78	4.2E-03	chr19q13.3-q13.4
zinc finger CCCH-type containing 12A	ZC3H12A	Hs.656294	7.75	3.9E-03	chr1p34.3
transient receptor potential cation channel, subfamily A, member 1	TRPA1	Hs.716816	7.65	2.1E-03	chr8q13
claudin 1	CLDN1	Hs.439060	7.45	3.8E-03	chr3q28-q29
BCL2-related protein A1	BCL2A1	Hs.227817	7.42	1.1E-02	chr15q24.3
kynureninase (L-kynurenine hydrolase)	KYNU	Hs.470126	7.34	7.5E-03	chr2q22.2
six transmembrane epithelial antigen of the prostate 2	STEAP2	Hs.489051	7.32	2.3E-03	chr7q21
tumor necrosis factor (ligand) superfamily, member 10	TNFSF10	Hs.478275	7.16	6.8E-03	chr3q26
2'-5'-oligoadenylate synthetase 2, 69/71kDa	OAS2	Hs.414332	7.09	6.8E-03	chr12q24.2

SM-Table 1: Genes Significantly Upregulated (>2 fold, p<0.05) in HTM Cells Phagocytically Challenged to E. coli Under Physiological Conditions

Gene Title	Gene Symbol	UniGene ID	Fold	PValue	Chromosomal Location
XIAP associated factor 1	XAF1	Hs.441975	6.63	1.2E-03	chr17p13.2
angiopoietin-like 1	ANGPTL1	Hs.591474	6.48	2.9E-03	chr1q25.2
ST3 beta-galactoside alpha-2,3-sialyltransferase 6	ST3GAL6	Hs.148716	6.36	5.0E-03	chr3q12.1
DEAD (Asp-Glu-Ala-Asp) box polypeptide 58	DDX58	Hs.190622	6.23	4.7E-03	chr9p12
interleukin 32	IL32	Hs.943	6.20	4.1E-03	chr16p13.3
DEAD (Asp-Glu-Ala-Asp) box polypeptide 60	DDX60	Hs.591710	6.10	1.9E-03	chr4q32.3
six transmembrane epithelial antigen of the prostate 1	STEAP1	Hs.61635	6.03	2.7E-03	chr7q21
insulin-like growth factor 1 (somatomedin C)	IGF1	Hs.160562	5.97	7.3E-03	chr12q22-q23
F-box protein 32	FBXO32	Hs.403933	5.62	4.6E-03	chr8q24.13
tripartite motif-containing 14	TRIM14	Hs.575631	5.54	2.9E-03	chr9q22.33
tumor necrosis factor, alpha-induced protein 2	TNFAIP2	Hs.525607	5.53	2.1E-03	chr14q32
hect domain and RLD 5	HERC5	Hs.26663	5.52	2.7E-03	chr4q22.1
interferon-induced protein with tetratricopeptide repeats 2	IFIT2	Hs.437609	5.47	3.8E-03	chr10q23-q25
bradykinin receptor B1	BDKRB1	Hs.525572	5.45	4.7E-03	chr14q32.1-q32.2
signal transducer and activator of transcription 1, 91kDa	STAT1	Hs.715518	5.43	2.1E-03	chr2q32.2
leukemia inhibitory factor (cholinergic differentiation factor)	LIF	Hs.2250	5.38	3.4E-03	chr22q12.2
chemokine (C-X-C motif) ligand 6 (granulocyte chemotactic protein 2)	CXCL6	Hs.164021	5.30	7.7E-03	chr4q21
nicotinamide phosphoribosyltransferase	NAMPT	Hs.489615	5.25	3.3E-03	chr7q22.2
nuclear factor (erythroid-derived 2)-like 3	NFE2L3	Hs.404741	5.07	6.0E-03	chr7p15-p14
229339_at		Hs.462257	4.93	4.6E-03	
interleukin-1 receptor-associated kinase 2	IRAK2	Hs.449207	4.90	6.4E-03	chr3p25.3
GTP cyclohydrolase 1	GCH1	Hs.86724	4.87	3.3E-03	chr14q22.1-q22.2
interferon regulatory factor 7	IRF7	Hs.166120	4.86	2.8E-03	chr11p15.5
tumor necrosis factor (ligand) superfamily, member 13b	TNFSF13B	Hs.525157	4.83	2.1E-03	chr13q32-q34
prostaglandin-endoperoxide synthase 2 (prostaglandin G/H synthase and cyclooxygenase)	PTGS2	Hs.196384	4.82	2.1E-03	chr1q25.2-q25.3
damage-regulated autophagy modulator	DRAM	Hs.525634	4.79	2.1E-03	chr12q23.2
intercellular adhesion molecule 1	ICAM1	Hs.643447	4.75	5.6E-03	chr19p13.3-p13.2
metallothionein 1F	MT1F	Hs.513626	4.73	6.5E-03	chr16q13
matrix metallopeptidase 1 (interstitial collagenase)	MMP1	Hs.83169	4.61	3.2E-03	chr11q22.3
zona pellucida glycoprotein 4	ZP4	Hs.136241	4.60	4.6E-03	chr1q43
endothelial cell-specific molecule 1	ESM1	Hs.129944	4.60	3.1E-02	chr5q11.2
delta/notch-like EGF repeat containing	DNER	Hs.234074	4.57	5.2E-03	chr2q36.3
vascular cell adhesion molecule 1	VCAM1	Hs.109225	4.43	2.1E-03	chr1p32-p31

SM-Table 1: Genes Significantly Upregulated (>2 fold, p<0.05) in HTM Cells Phagocytically Challenged to E. coli Under Physiological Conditions

Gene Title	Gene Symbol	UniGene ID	Fold	PValue	Chromosomal Location
bone morphogenetic protein 2	BMP2	Hs.73853	4.43	2.5E-03	chr20p12
phospholipid scramblase 1	PLSCR1	Hs.130759	4.40	2.8E-03	chr3q23
nuclear factor of kappa light polypeptide gene enhancer in B-cells inhibitor, alpha	NFKBIA	Hs.81328	4.39	2.1E-03	chr14q13
signal transducer and activator of transcription 4	STAT4	Hs.80642	4.39	6.1E-03	chr2q32.2-q32.3
5-hydroxytryptamine (serotonin) receptor 2A	HTR2A	Hs.654586	4.35	4.7E-03	chr13q14-q21
interferon-induced protein 35	IFI35	Hs.632258	4.25	5.8E-03	chr17q21
similar to hCG38149 /// ovostatin /// ovostatin 2	LOC728715 /	Hs.568152	4.19	3.9E-03	chr12p11.21 /// chr1
PR domain containing 1, with ZNF domain	PRDM1	Hs.436023	4.19	8.4E-03	chr6q21-q22.1
adenosine monophosphate deaminase (isoform E)	AMPD3	Hs.501890	4.19	2.5E-03	chr11p15
2'-5'-oligoadenylate synthetase-like	OASL	Hs.118633	4.16	4.7E-03	chr12q24.2
interferon-induced protein 44	IFI44	Hs.82316	4.15	2.1E-03	chr1p31.1
vanin 3	VNN3	Hs.183656	4.13	4.9E-03	chr6q23-q24
solute carrier family 39 (zinc transporter), member 14	SLC39A14	Hs.491232	4.13	4.1E-03	chr8p21.3
indoleamine 2,3-dioxygenase 1	IDO1	Hs.840	4.11	1.2E-02	chr8p12-p11
regulator of calcineurin 1	RCAN1	Hs.282326	4.01	6.0E-03	chr21q22.1-q22.2|21
Norrie disease (pseudoglioma)	NDP	Hs.522615	3.90	1.4E-02	chrXp11.4
deiodinase, iodothyronine, type II	DIO2	Hs.202354	3.83	3.7E-03	chr14q24.2-q24.3
interleukin-1 receptor-associated kinase 3	IRAK3	Hs.369265	3.83	1.0E-02	chr12q14.3
interleukin 18 binding protein	IL18BP	Hs.591967	3.82	2.0E-03	chr11q13
chitinase 3-like 1 (cartilage glycoprotein-39)	CHI3L1	Hs.382202	3.82	8.8E-03	chr1q32.1
retinoic acid receptor responder (tazarotene induced) 1	RARRES1	Hs.131269	3.72	7.9E-03	chr3q25.32-q25.33
v-maf musculoaponeurotic fibrosarcoma oncogene homolog F (avian)	MAFF	Hs.517617	3.71	3.9E-03	chr22q13.1
235438_at		Hs.657330	3.70	4.2E-02	
sclerosteosis	SOST	Hs.349204	3.67	1.7E-02	chr17q11.2
toll-like receptor 2	TLR2	Hs.519033	3.67	1.9E-03	chr4q32
ABI family, member 3 (NESH) binding protein	ABI3BP	Hs.477015	3.65	5.1E-03	chr3q12
tumor necrosis factor (ligand) superfamily, member 18	TNFSF18	Hs.248197	3.65	7.2E-03	chr1q23
solute carrier family 15, member 3	SLC15A3	Hs.237856	3.61	5.5E-03	chr11q12.2
nuclear receptor subfamily 4, group A, member 2	NR4A2	Hs.563344	3.60	3.5E-03	chr2q22-q23
interferon, gamma-inducible protein 30	IFI30	Hs.14623	3.59	2.5E-03	chr19p13.1
histone cluster 2, H2aa3 /// histone cluster 2, H2aa4	HIST2H2AA3	Hs.530461	3.57	1.4E-02	chr1q21.2
sterile alpha motif domain containing 9-like	SAMD9L	Hs.489118	3.56	2.3E-03	chr7q21.2-q21.3
v-yes-1 Yamaguchi sarcoma viral related oncogene homolog	LYN	Hs.699154	3.54	6.8E-03	chr8q13

SM-Table 1: Genes Significantly Upregulated (>2 fold, p<0.05) in HTM Cells Phagocytically Challenged to E. coli Under Physiological Conditions

Gene Title	Gene Symbol	UniGene ID	Fold	PValue	Chromosomal Location
poly (ADP-ribose) polymerase family, member 12	PARP12	Hs.12646	3.54	2.2E-03	chr7q34
spermidine/spermine N1-acetyltransferase 1	SAT1	Hs.28491	3.51	4.2E-03	chrXp22.1
244130_at		Hs.72630	3.47	4.3E-03	
poly (ADP-ribose) polymerase family, member 14	PARP14	Hs.518203	3.44	3.2E-03	chr3q21.1
pigeon homolog (Drosophila)	PION	Hs.186649	3.42	7.5E-03	chr7q11.23
solute carrier family 11 (proton-coupled divalent metal ion transporters), member 2	SLC11A2	Hs.505545	3.42	1.8E-03	chr12q13
matrix metallopeptidase 8 (neutrophil collagenase)	MMP8	Hs.161839	3.39	5.7E-03	chr11q22.3
Epithelial stromal interaction 1 (breast)	EPSTI1	Hs.546467	3.38	2.1E-03	chr13q13.3
Wilms tumor 1 associated protein	WTAP	Hs.446091	3.36	3.4E-03	chr6q25-q27
sperm associated antigen 4	SPAG4	Hs.123159	3.36	8.2E-03	chr20q11.21
interleukin 1, alpha	IL1A	Hs.1722	3.36	2.7E-03	chr2q14
sterile alpha motif domain containing 9	SAMD9	Hs.65641	3.34	2.1E-03	chr7q21.2
ubiquitin specific peptidase 18	USP18	Hs.38260	3.34	7.1E-03	chr22q11.21
nuclear receptor subfamily 4, group A, member 3	NR4A3	Hs.279522	3.33	3.8E-03	chr9q22
v-rel reticuloendotheliosis viral oncogene homolog B	RELB	Hs.654402	3.32	9.1E-03	chr19q13.32
interferon induced transmembrane protein 1 (9-27)	IFITM1	Hs.458414	3.31	3.0E-03	chr11p15.5
ELOVL family member 7, elongation of long chain fatty acids (yeast)	ELOVL7	Hs.274256	3.30	4.9E-03	chr5q12.1
Uncharacterized protein LOC100131897	LOC1001318	Hs.659330	3.30	5.0E-03	chr5q35.1
metallothionein 1G	MT1G	Hs.433391	3.30	5.7E-03	chr16q13
cholesterol 25-hydroxylase	CH25H	Hs.47357	3.29	2.1E-03	chr10q23
solute carrier family 19, member 3	SLC19A3	Hs.221597	3.27	4.1E-03	chr2q37
membrane-associated ring finger (C3HC4) 3	MARCH3	Hs.132441	3.27	6.1E-03	chr5q23.2
serpin peptidase inhibitor, clade E (nexin, plasminogen activator inhibitor type 1), member 1	SERPINE1	Hs.414795	3.17	3.7E-03	chr7q21.3-q22
receptor (chemosensory) transporter protein 4	RTP4	Hs.43388	3.15	2.3E-03	chr3q27.3
epiregulin	EREG	Hs.115263	3.14	2.3E-02	chr4q13.3
ring finger protein 144B	RNF144B	Hs.148741	3.14	2.7E-03	chr6p22.3
cingulin-like 1	CGNL1	Hs.148989	3.14	1.1E-02	chr15q21.3
family with sequence similarity 65, member C	FAM65C	Hs.372578	3.13	5.3E-03	chr20q13.13
chromosome 6 open reading frame 176	C6orf176	Hs.31917	3.13	3.7E-03	chr6q27
CCAAT/enhancer binding protein (C/EBP), delta	CEBPD	Hs.440829	3.12	5.2E-03	chr8p11.2-p11.1
tumor necrosis factor receptor superfamily, member 11b	TNFRSF11B	Hs.81791	3.12	1.8E-03	chr8q24
KIAA0146	KIAA0146	Hs.381058	3.12	3.9E-03	chr8q11.21
neuronal cell adhesion molecule	NRCAM	Hs.21422	3.11	2.8E-03	chr7q31.1-q31.2

SM-Table 1: Genes Significantly Upregulated (>2 fold, p<0.05) in HTM Cells Phagocytically Challenged to E. coli Under Physiological Conditions

Gene Title	Gene Symbol	UniGene ID	Fold	PValue	Chromosomal Location
metallothionein 1X	MT1X	Hs.374950	3.10	9.4E-03	chr16q13
carbonic anhydrase XII	CA12	Hs.210995	3.09	3.9E-03	chr15q22
proteasome (prosome, macropain) subunit, beta type, 9 (large multifunctional peptidase 2)	PSMB9	Hs.654585	3.04	1.1E-02	chr6p21.3
nuclear factor of kappa light polypeptide gene enhancer in B-cells 2 (p49/p100)	NFKB2	Hs.73090	3.03	2.5E-03	chr10q24
225328_at		Hs.592775	3.02	2.1E-03	
acyl-CoA synthetase long-chain family member 4	ACSL4	Hs.268785	3.01	9.1E-03	chrXq22.3-q23
210524_x_at			3.01	1.1E-02	
BCL2/adenovirus E1B 19kDa interacting protein 3	BNIP3	Hs.144873	2.98	6.7E-03	chr10q26.3
dipeptidyl-peptidase 4	DPP4	Hs.368912	2.98	8.2E-03	chr2q24.3
potassium inwardly-rectifying channel, subfamily J, member 15	KCNJ15	Hs.411299	2.96	3.1E-03	chr21q22.2
podoplanin	PDPN	Hs.468675	2.96	1.8E-03	chr1p36.21
thymidine phosphorylase	TYMP	Hs.592212	2.92	2.9E-03	chr22q13|22q13.33
leucine aminopeptidase 3	LAP3	Hs.570791	2.91	7.6E-03	chr4p15.32
R-spondin 3 homolog (Xenopus laevis)	RSPO3	Hs.135254	2.90	2.3E-03	chr6q22.33
caspase recruitment domain family, member 16	CARD16	Hs.348365	2.83	5.8E-03	
piccolo (presynaptic cytomatrix protein)	PCLO	Hs.12376	2.81	1.0E-02	chr7q11.23-q21.3
metallothionein 1H	MT1H	Hs.438462	2.78	8.4E-03	chr16q13
neurofilament, medium polypeptide	NEFM	Hs.458657	2.77	2.1E-02	chr8p21
metallothionein 1E /// metallothionein 1H /// metallothionein 1M /// metallothionein 1 pseudogene 2	MT1E /// MT1	Hs.534330	2.77	5.1E-03	chr16q13 /// chr1q43
betacellulin	BTC	Hs.710156	2.75	2.4E-02	chr4q13-q21
DEAD (Asp-Glu-Ala-Asp) box polypeptide 60-like	DDX60L	Hs.535011	2.75	8.4E-03	chr4q32.3
proteasome (prosome, macropain) activator subunit 2 (PA28 beta)	PSME2	Hs.434081	2.74	2.9E-03	chr14q11.2
RAS, dexamethasone-induced 1	RASD1	Hs.25829	2.73	1.4E-02	chr17p11.2
tumor necrosis factor (ligand) superfamily, member 11	TNFSF11	Hs.333791	2.72	5.2E-03	chr13q14
229802_at		Hs.593316	2.72	6.3E-03	
outer dense fiber of sperm tails 3B	ODF3B	Hs.531314	2.71	3.5E-03	chr22q13.33
227458_at		Hs.655937	2.68	5.1E-03	
vascular endothelial growth factor C	VEGFC	Hs.435215	2.67	1.9E-03	chr4q34.1-q34.3
Meis homeobox 1	MEIS1	Hs.526754	2.66	1.2E-02	chr2p14-p13
244219_at		Hs.658454	2.66	4.6E-03	
secreted frizzled-related protein 2	SFRP2	Hs.481022	2.65	9.4E-03	chr4q31.3

SM-Table 1: Genes Significantly Upregulated (>2 fold, p<0.05) in HTM Cells Phagocytically Challenged to E. coli Under Physiological Conditions

Gene Title	Gene Symbol	UniGene ID	Fold	PValue	Chromosomal Location
PDZ domain containing 2	PDZD2	Hs.481819	2.65	1.2E-02	chr5p13.3
solute carrier family 16, member 3 (monocarboxylic acid transporter 4)	SLC16A3	Hs.500761	2.64	3.4E-03	chr17q25
spermatogenesis associated 13	SPATA13	Hs.657121	2.64	3.8E-03	chr13q12.12
ADAM metallopeptidase domain 28	ADAM28	Hs.174030	2.64	4.2E-02	chr8p21.2
interleukin 1, beta	IL1B	Hs.126256	2.64	1.8E-02	chr2q14
integrin, alpha 2 (CD49B, alpha 2 subunit of VLA-2 receptor)	ITGA2	Hs.482077	2.64	7.5E-03	chr5q23-q31
transmembrane protein 140	TMEM140	Hs.719309	2.64	1.3E-02	chr7q33
kinase insert domain receptor (a type III receptor tyrosine kinase)	KDR	Hs.479756	2.63	1.1E-02	chr4q11-q12
suppressor of cytokine signaling 2	SOCS2	Hs.485572	2.63	5.2E-03	chr12q
tripartite motif-containing 22	TRIM22	Hs.501778	2.63	3.8E-03	chr11p15
1555852_at			2.62	1.2E-02	
calcium binding protein 1	CABP1	Hs.458482	2.62	2.2E-02	chr12q24.31
major histocompatibility complex, class I, B	HLA-B	Hs.703277	2.61	2.5E-03	chr6p21.3
hypothetical protein LOC100132999	LOC1001329	Hs.670106	2.61	5.1E-03	chr1q21.1
transporter 1, ATP-binding cassette, sub-family B (MDR/TAP)	TAP1	Hs.352018	2.61	4.9E-03	chr6p21.3
interferon stimulated exonuclease gene 20kDa	ISG20	Hs.459265	2.57	7.9E-03	chr15q26
aldehyde oxidase 1	AOX1	Hs.406238	2.57	1.1E-02	chr2q33
cytochrome P450, family 7, subfamily B, polypeptide 1	CYP7B1	Hs.667720	2.57	5.6E-03	chr8q21.3
239587_at		Hs.201600	2.57	3.4E-03	
methylenetetrahydrofolate dehydrogenase (NADP+ dependent) 2-like	MTHFD2L	Hs.479954	2.56	2.1E-03	chr4q13.3
solute carrier family 43, member 3	SLC43A3	Hs.99962	2.56	2.1E-03	chr11q11
lectin, galactoside-binding, soluble, 3 binding protein /// similar to lectin, galactoside-binding, soluble, 3 binding protein	LGALS3BP //	Hs.514535	2.56	1.9E-03	chr17q25
peroxisomal proliferator-activated receptor A interacting complex 285	PRIC285	Hs.517180	2.55	1.1E-02	chr20q13.33
metallothionein 1 pseudogene 2	MT1P2	Hs.632513	2.55	7.2E-03	chr1q43
neuronal PAS domain protein 2	NPAS2	Hs.156832	2.55	4.2E-03	chr2q11.2
bradykinin receptor B2	BDKRB2	Hs.719484	2.55	6.2E-03	chr14q32.1-q32.2
major histocompatibility complex, class I, F	HLA-F	Hs.519972	2.54	2.5E-03	chr6p21.3
232712_at		Hs.248049	2.54	7.5E-03	

SM-Table 1: Genes Significantly Upregulated (>2 fold, p<0.05) in HTM Cells Phagocytically Challenged to E. coli Under Physiological Conditions

Gene Title	Gene Symbol	UniGene ID	Fold	PValue	Chromosomal Location
230333_at		Hs.656630	2.54	1.4E-02	
G0/G1switch 2	G0S2	Hs.432132	2.53	7.8E-03	chr1q32.2|1q32.2-q4
GTP binding protein overexpressed in skeletal muscle	GEM	Hs.654463	2.53	1.2E-02	chr8q13-q21
NK3 homeobox 1	NKX3-1	Hs.55999	2.51	1.5E-02	chr8p21
immediate early response 3	IER3	Hs.591785	2.50	4.9E-03	chr6p21.3
hyaluronan synthase 2	HAS2	Hs.159226	2.50	7.9E-03	chr8q24.12
caspase 1, apoptosis-related cysteine peptidase (interleukin 1, beta, convertase)	CASP1	Hs.2490	2.49	5.7E-03	chr11q23
signal-regulatory protein alpha	SIRPA	Hs.581021	2.49	5.3E-03	chr20p13
6-phosphofructo-2-kinase/fructose-2,6-biphosphatase 4	PFKFB4	Hs.476217	2.49	5.2E-03	chr3p22-p21
heat shock 27kDa protein 3	HSPB3	Hs.41707	2.48	2.1E-03	chr5q11.2
agrin	AGRN	Hs.273330	2.48	4.9E-03	chr1p36.33
ubiquitin-conjugating enzyme E2L 6	UBE2L6	Hs.425777	2.47	7.9E-03	chr11q12
chromosome 7 open reading frame 68	C7orf68	Hs.710088	2.47	5.3E-03	chr7q32.1
proline-serine-threonine phosphatase interacting protein 2	PSTPIP2	Hs.567384	2.46	6.3E-03	chr18q12
zinc finger CCCH-type containing 12C	ZC3H12C	Hs.376289	2.46	1.4E-02	chr11q22.3
metallothionein 2A	MT2A	Hs.647371	2.46	7.7E-03	chr16q13
chromosome 1 open reading frame 38	C1orf38	Hs.10649	2.46	1.5E-02	chr1p35.3
gremlin 1, cysteine knot superfamily, homolog (Xenopus laevis)	GREM1	Hs.40098	2.45	1.9E-03	chr15q13-q15
acyl-CoA synthetase long-chain family member 5	ACSL5	Hs.11638	2.45	7.4E-03	chr10q25.1-q25.2
chromosome 13 open reading frame 33	C13orf33	Hs.646647	2.44	9.5E-03	chr13q12.3
interleukin 1 receptor antagonist	IL1RN	Hs.81134	2.44	2.1E-03	chr2q14.2
GATA binding protein 6	GATA6	Hs.514746	2.44	5.0E-03	chr18q11.1-q11.2
EF-hand calcium binding domain 3 /// similar to hypoxia-inducible protein 2	EFCAB3 /// L	Hs.152670	2.43	3.4E-03	chr17q23.2
musculin (activated B-cell factor-1)	MSC	Hs.442619	2.43	5.7E-03	chr8q21
242907_at			2.43	6.9E-03	
TNFAIP3 interacting protein 1	TNIP1	Hs.355141	2.43	1.0E-02	chr5q32-q33.1
interleukin 15 receptor, alpha	IL15RA	Hs.524117	2.43	8.7E-03	chr10p15-p14
CD82 molecule	CD82	Hs.527778	2.42	1.9E-03	chr11p11.2
major histocompatibility complex, class I, C	HLA-C	Hs.654404	2.41	7.8E-03	chr6p21.3
colony stimulating factor 3 (granulocyte)	CSF3	Hs.2233	2.41	7.6E-03	chr17q11.2-q12
interferon regulatory factor 9	IRF9	Hs.1706	2.40	7.6E-03	chr14q11.2
tumor necrosis factor receptor superfamily, member 21	TNFRSF21	Hs.443577	2.40	1.2E-02	chr6p21.1-p12.2

SM-Table 1: Genes Significantly Upregulated (>2 fold, p<0.05) in HTM Cells Phagocytically Challenged to E. coli Under Physiological Conditions

Gene Title	Gene Symbol	UniGene ID	Fold	PValue	Chromosomal Location
HECT, C2 and WW domain containing E3 ubiquitin protein ligase 2	HECW2	Hs.654742	2.40	2.2E-03	chr2q32.3-q33.1
dystrobrevin, alpha	DTNA	Hs.643454	2.40	1.1E-02	chr18q12
pleckstrin homology-like domain, family A, member 1	PHLDA1	Hs.602085	2.39	2.9E-03	chr12q15
C-type lectin domain family 2, member B	CLEC2B	Hs.85201	2.38	1.1E-02	chr12p13-p12
solute carrier family 22, member 23	SLC22A23	Hs.713588	2.38	2.8E-03	chr6p25.2
platelet factor 4 variant 1	PF4V1	Hs.72933	2.38	1.5E-02	chr4q12-q21
RAS guanyl releasing protein 1 (calcium and DAG-regulated)	RASGRP1	Hs.591127	2.37	2.1E-03	chr15q14
adenylate kinase 3-like 1	AK3L1	Hs.592601	2.37	4.1E-03	chr1p31.3
LIM and cysteine-rich domains 1	LMCD1	Hs.475353	2.37	1.2E-02	chr3p26-p24
poly (ADP-ribose) polymerase family, member 9	PARP9	Hs.518200	2.37	6.9E-03	chr3q21
C1q and tumor necrosis factor related protein 1	C1QTNF1	Hs.201398	2.36	1.0E-02	chr17q25.3
apolipoprotein B mRNA editing enzyme, catalytic polypeptide-like 3G	APOBEC3G	Hs.660143	2.35	1.1E-02	chr22q13.1-q13.2
endoplasmic reticulum aminopeptidase 2	ERAP2	Hs.482910	2.35	2.6E-02	chr5q15
G protein-coupled receptor, family C, group 5, member B	GPRC5B	Hs.148685	2.35	4.2E-03	chr16p12
mannosidase, alpha, class 1A, member 1	MAN1A1	Hs.102788	2.35	8.3E-03	chr6q22
early growth response 2	EGR2	Hs.1395	2.34	2.3E-03	chr10q21.1
hypothetical protein FLJ36031	FLJ36031	Hs.29692	2.33	1.1E-02	chr7q22.3
chemokine (C-X-C motif) ligand 16	CXCL16	Hs.711652	2.33	3.5E-03	chr17p13
tripartite motif-containing 69	TRIM69	Hs.489254	2.33	6.8E-03	chr15q21.1
gap junction protein, delta 3, 31.9kDa	GJD3	Hs.444663	2.33	2.7E-02	chr17q21.2
236480_at		Hs.446388	2.33	3.9E-03	
lipoprotein lipase	LPL	Hs.180878	2.32	8.7E-03	chr8p22
deltex 3-like (Drosophila)	DTX3L	Hs.518201	2.32	4.5E-03	chr3q21.1
egl nine homolog 3 (C. elegans)	EGLN3	Hs.135507	2.32	2.4E-02	chr14q13.1
fibroblast growth factor 10	FGF10	Hs.664499	2.31	1.6E-02	chr5p13-p12
serglycin	SRGN	Hs.1908	2.31	4.1E-03	chr10q22.1
pannexin 1	PANX1	Hs.591976	2.31	2.9E-03	chr11q21
glycoprotein M6B	GPM6B	Hs.495710	2.31	6.5E-03	chrXp22.2
toll-like receptor 1	TLR1	Hs.654532	2.31	6.4E-03	chr4p14
235661_at		Hs.649101	2.30	3.9E-02	
similar to WDNM1-like protein	LOC645638	Hs.463652	2.30	6.7E-03	chr17q23.1
family with sequence similarity 20, member A	FAM20A	Hs.268874	2.30	7.5E-03	chr17q24.2
oxidized low density lipoprotein (lectin-like) receptor 1	OLR1	Hs.412484	2.30	2.5E-02	chr12p13.2-p12.3

SM-Table 1: Genes Significantly Upregulated (>2 fold, p<0.05) in HTM Cells Phagocytically Challenged to E. coli Under Physiological Conditions

Gene Title	Gene Symbol	UniGene ID	Fold	PValue	Chromosomal Location
prostaglandin E synthase	PTGES	Hs.146688	2.29	9.3E-03	chr9q34.3
sorbin and SH3 domain containing 2	SORBS2	Hs.655143	2.29	5.2E-03	chr4q35.1
twist homolog 2 (Drosophila)	TWIST2	Hs.422585	2.28	1.3E-02	chr2q37.3
guanylate binding protein 1, interferon-inducible, 67kDa	GBP1	Hs.62661	2.28	3.3E-03	chr1p22.2
procollagen-lysine, 2-oxoglutarate 5-dioxygenase 2	PLOD2	Hs.477866	2.27	6.7E-03	chr3q23-q24
regulator of G-protein signaling 3	RGS3	Hs.494875	2.27	6.6E-03	chr9q32
toll-like receptor 3	TLR3	Hs.657724	2.27	8.4E-03	chr4q35
protein kinase (cAMP-dependent, catalytic) inhibitor beta	PKIB	Hs.719228	2.26	5.8E-03	chr6q22.31
prostaglandin I2 (prostacyclin) synthase	PTGIS	Hs.302085	2.26	1.8E-02	chr20q13.13
chemokine (C-X-C motif) ligand 9	CXCL9	Hs.77367	2.26	3.9E-03	chr4q21
colony stimulating factor 1 (macrophage)	CSF1	Hs.591402	2.26	1.9E-03	chr1p21-p13
tyrosinase-related protein 1	TYRP1	Hs.270279	2.25	4.6E-03	chr9p23
interleukin 7 receptor	IL7R	Hs.635723	2.24	7.1E-03	chr5p13
transporter 2, ATP-binding cassette, sub-family B (MDR/TAP)	TAP2	Hs.502	2.24	2.1E-03	chr6p21.3
butyrophilin, subfamily 3, member A2	BTN3A2	Hs.376046	2.23	1.9E-03	chr6p22.1
histone cluster 1, H2bc	HIST1H2BC	Hs.658713	2.23	1.0E-02	chr6p21.3
solute carrier family 7 (cationic amino acid transporter, y+ system), member 2	SLC7A2	Hs.448520	2.23	2.1E-03	chr8p22-p21.3
hypothetical protein LOC285628	LOC285628	Hs.604728	2.23	3.1E-03	chr5q34
phosphoenolpyruvate carboxykinase 1 (soluble)	PCK1	Hs.1872	2.23	2.8E-02	chr20q13.31
major histocompatibility complex, class I, E	HLA-E	Hs.650174	2.23	6.1E-03	chr6p21.3
tumor-associated calcium signal transducer 2	TACSTD2	Hs.23582	2.22	1.4E-02	chr1p32-p31
basic leucine zipper transcription factor, ATF-like 2	BATF2	Hs.124840	2.22	1.4E-02	chr11q13.1
phospholipase A2, group IVA (cytosolic, calcium-dependent)	PLA2G4A	Hs.497200	2.21	1.3E-02	chr1q25
dual specificity phosphatase 4	DUSP4	Hs.417962	2.21	7.2E-03	chr8p12-p11
transmembrane protein 132B	TMEM132B	Hs.524838	2.21	2.8E-02	chr12q24.31-q24.32
214124_x_at		Hs.597151	2.21	2.1E-03	
chromosome 11 open reading frame 17 /// NUAK family, SNF1-like kinase, 2	C11orf17 /// N	Hs.131180	2.21	4.1E-03	chr11p15.3 /// chr1q
histone cluster 2, H2be	HIST2H2BE	Hs.2178	2.20	7.3E-03	chr1q21-q23
BMP binding endothelial regulator	BMPER	Hs.660998	2.20	5.2E-03	chr7p14.3
238743_at		Hs.546523	2.19	2.4E-03	
family with sequence similarity 26, member F	FAM26F	Hs.381220	2.19	1.2E-02	chr6q22.1

SM-Table 1: Genes Significantly Upregulated (>2 fold, p<0.05) in HTM Cells Phagocytically Challenged to E. coli Under Physiological Conditions

Gene Title	Gene Symbol	UniGene ID	Fold	PValue	Chromosomal Location
phospholipase A2, group IVC (cytosolic, calcium-independent)	PLA2G4C	Hs.631562	2.19	2.4E-02	chr19q13.3
microRNA host gene 2 (non-protein coding)	MIRHG2	Hs.697120	2.18	2.1E-03	
Pelota homolog (Drosophila)	PELO	Hs.644352	2.18	1.9E-02	chr5q11.2
tribbles homolog 1 (Drosophila)	TRIB1	Hs.444947	2.17	2.4E-03	chr8q24.13
family with sequence similarity 162, member A	FAM162A	Hs.584881	2.17	1.2E-02	chr3q21.1
major histocompatibility complex, class I, A /// major histocompatibility complex, class I, B /// major histocompatibility complex, class I, G /// major histocompatibility complex, class I, H (pseudogene) /// major histocompatibility complex, class I, J (pseudogene)	HLA-A /// HL/	Hs.181244	2.16	1.8E-03	chr6p21.3 /// chr6p2
glycerol kinase	GK	Hs.1466	2.16	1.6E-02	chrXp21.3
230741_at		Hs.439682	2.15	9.1E-03	
proprotein convertase subtilisin/kexin type 1	PCSK1	Hs.78977	2.14	2.3E-02	chr5q15-q21
paroxysmal nonkinesigenic dyskinesia	PNKD	Hs.98475	2.14	4.3E-03	chr2q35
sidekick homolog 1, cell adhesion molecule (chicken)	SDK1	Hs.653013	2.13	3.4E-02	chr7p22.2
dual specificity phosphatase 6	DUSP6	Hs.298654	2.13	1.1E-02	chr12q22-q23
SP100 nuclear antigen	SP100	Hs.369056	2.13	5.1E-03	chr2q37.1
UDP-glucose ceramide glucosyltransferase	UGCG	Hs.304249	2.12	8.6E-03	chr9q31
inhibin, beta A	INHBA	Hs.583348	2.12	6.7E-03	chr7p15-p13
227621_at		Hs.708272	2.12	5.7E-03	
plasminogen activator, urokinase receptor	PLAUR	Hs.466871	2.11	9.2E-03	chr19q13
neuronal PAS domain protein 2	NPAS2	Hs.156832	2.11	1.9E-02	chr2q11.2
HLA complex P5	HCP5	Hs.654480	2.10	4.4E-02	chr6p21.3
piwi-like 4 (Drosophila)	PIWIL4		2.10	1.1E-02	chr11q21
POU class 2 homeobox 2	POU2F2	Hs.654420	2.10	4.8E-03	chr19q13.2
1558365_at		Hs.605605	2.09	1.6E-02	
ATP-binding cassette, sub-family A (ABC1), member 6	ABCA6	Hs.709514	2.09	3.4E-02	chr17q24.3
227140_at		Hs.28792	2.08	1.8E-03	
pregnancy-associated plasma protein A, pappalysin 1	PAPPA	Hs.643599	2.08	1.4E-02	chr9q33.2
junctional adhesion molecule 2	JAM2	Hs.517227	2.08	5.0E-03	chr21q21.2
aryl hydrocarbon receptor nuclear translocator-like 2	ARNTL2	Hs.434269	2.07	1.3E-02	chr12p12.2-p11.2
synuclein, alpha interacting protein	SNCAIP	Hs.426463	2.07	2.8E-03	chr5q23.1-q23.3
coagulation factor III (thromboplastin, tissue factor)	F3	Hs.62192	2.07	1.2E-02	chr1p22-p21
FOS-like antigen 1	FOSL1	Hs.283565	2.07	9.3E-03	chr11q13

SM-Table 1: Genes Significantly Upregulated (>2 fold, p<0.05) in HTM Cells Phagocytically Challenged to E. coli Under Physiological Conditions

Gene Title	Gene Symbol	UniGene ID	Fold	PValue	Chromosomal Location
237365_at		Hs.43818	2.07	5.5E-03	
Hypothetical protein LOC100128718	LOC1001287	Hs.707915	2.06	4.6E-03	chr19p13.11
interferon-induced protein with tetratricopeptide repeats 5	IFIT5	Hs.252839	2.06	3.2E-03	chr10q23.31
chromosome 6 open reading frame 192	C6orf192	Hs.347144	2.06	1.3E-03	chr6q22.3-q23.3
major histocompatibility complex, class I, G	HLA-G	Hs.512152	2.06	4.2E-03	chr6p21.3
ATPase type 13A3	ATP13A3	Hs.529609	2.06	3.8E-03	chr3q29
solute carrier family 2 (facilitated glucose transporter), member 6	SLC2A6	Hs.244378	2.05	9.1E-03	chr9q34
chromosome 7 open reading frame 58	C7orf58	Hs.189652	2.05	2.7E-03	chr7q31.31
CD38 molecule	CD38	Hs.479214	2.05	2.2E-02	chr4p15
interleukin 18 receptor 1	IL18R1	Hs.469521	2.05	1.2E-02	chr2q12
chromosome 19 open reading frame 66	C19orf66	Hs.655613	2.05	1.3E-02	chr19p13.2
eukaryotic translation initiation factor 2-alpha kinase 2	EIF2AK2	Hs.131431	2.05	1.7E-02	chr2p22-p21
solute carrier family 16, member 6 (monocarboxylic acid transporter 7)	SLC16A6	Hs.42645	2.04	4.0E-02	chr17q24.2
protein tyrosine phosphatase, non-receptor type 12	PTPN12	Hs.61812	2.04	1.4E-02	chr7q11.23
pyruvate dehydrogenase kinase, isozyme 1	PDK1	Hs.470633	2.04	3.9E-03	chr2q31.1
RNA binding motif protein 47	RBM47	Hs.518727	2.04	1.7E-02	chr4p13-p12
Teashirt zinc finger homeobox 2	TSHZ2	Hs.649877	2.03	2.9E-02	chr20q13.2
glycerol kinase 3 pseudogene	GK3P	Hs.654557	2.03	4.6E-02	chr4q32.1
leucine rich repeat neuronal 3	LRRN3	Hs.3781	2.03	4.6E-03	chr7q31.1
229242_at		Hs.23349	2.03	1.8E-02	
TAP binding protein (tapasin)	TAPBP	Hs.714746	2.03	5.1E-03	chr6p21.3
glycogen synthase 1 (muscle)	GYS1	Hs.386225	2.02	5.1E-03	chr19q13.3
solute carrier family 16, member 4 (monocarboxylic acid transporter 5)	SLC16A4	Hs.351306	2.02	1.2E-02	chr1p13.3
guanylate binding protein 2, interferon-inducible	GBP2	Hs.386567	2.02	2.5E-03	chr1p22.2
spondin 1, extracellular matrix protein	SPON1	Hs.705394	2.01	4.0E-02	chr11p15.2
N-myc (and STAT) interactor	NMI	Hs.54483	2.01	6.2E-03	chr2q23
FYVE, RhoGEF and PH domain containing 4	FGD4	Hs.117835	2.01	1.1E-02	chr12p11.21
ubiquitin-like modifier activating enzyme 7	UBA7	Hs.16695	2.01	4.4E-03	chr3p21
mixed lineage kinase domain-like	MLKL	Hs.119878	2.00	5.2E-03	chr16q22.3
guanylate binding protein 4	GBP4	Hs.409925	2.00	1.3E-02	chr1p22.2
lanosterol synthase (2,3-oxidosqualene-lanosterol cyclase)	LSS	Hs.596543	2.00	2.7E-02	chr21q22.3
