# Supplementary material for: Up-Regulated Expression of Extracellular Matrix Remodeling Genes in Phagocytically Challenged Trabecular Meshwork Cells
Source: PLoS One. 2012 Apr 18;7(4):e34792. doi: 10.1371/journal.pone.0034792 (PMC3329506; doi:10.1371/journal.pone.0034792)
Supplement: Table S2 — List of the genes significantly downregulated (>2 fold, p<0.05) in HTM Cells phagocytically challenged to E. coli under physiological conditions. Confluent cultures of HTM cells were grown for two weeks under physiological 5% O2 atmosphere, and then phagocytically challenged to E. coli bioparticles. Changes in gene expression at day 3 post-phagocytic challenge were evaluated by gene array using Affymetrix Human Genome U133 Plus 2.0 chips, and analyzed by Genespring Software. (PDF) [file pone.0034792.s003.pdf]

SM-Table 2: Genes Significantly Downregulated (>2 fold, p<0.05) in HTM Cells Phagocytically Challenged to E. coli Under Physiological Conditions

Gene Title	Gene Symbol	UniGene ID	Fold	PValue	Chromosomal Location
endothelin 3	EDN3	Hs.1408	5.78	4.1E-03	chr20q13.2-q13.3
228740_at		Hs.26766	5.73	4.5E-03	
tumor necrosis factor receptor superfamily, member 10d, decoy with truncated death domain	TNFRSF10D	Hs.213467	4.71	3.4E-03	chr8p21
Fibroblast growth factor 18	FGF18	Hs.87191	4.60	2.9E-03	chr5q34
ceroid-lipofuscinosis, neuronal 8 (epilepsy, progressive with mental retardation)	CLN8	Hs.127675	4.24	3.8E-03	chr8p23
H19, imprinted maternally expressed transcript (non-protein coding)	H19	Hs.533566	4.04	2.3E-03	chr11p15.5
sclerostin domain containing 1	SOSTDC1	Hs.648106	4.02	7.8E-03	chr7p21.1
AF4/FMR2 family, member 3	AFF3	Hs.444414	3.98	1.1E-02	chr2q11.2-q12
C1q and tumor necrosis factor related protein 7	C1QTNF7	Hs.153714	3.57	6.5E-03	chr4p16-p15
nanos homolog 1 (Drosophila)	NANOS1	Hs.591918	3.41	6.9E-03	chr10q26.11
ribosomal protein L27a	RPL27A	Hs.523463	3.25	1.7E-02	chr11p15
EPH receptor A3	EPHA3	Hs.123642	3.07	5.1E-03	chr3p11.2
aquaporin 1 (Colton blood group)	AQP1	Hs.76152	3.07	2.9E-02	chr7p14
RAB39B, member RAS oncogene family	RAB39B	Hs.632832	3.02	1.0E-02	chrXq28
G protein-coupled receptor 37 (endothelin receptor type B-like)	GPR37	Hs.406094	2.99	1.3E-02	chr7q31
238919_at		Hs.656886	2.98	2.2E-02	
interleukin 17 receptor D	IL17RD	Hs.150725	2.97	2.1E-02	chr3p14.3
solute carrier family 16, member 9 (monocarboxylic acid transporter 9)	SLC16A9	Hs.499709	2.97	2.1E-03	chr10q21.1
odd-skipped related 2 (Drosophila)	OSR2	Hs.253247	2.89	3.9E-03	chr8q22.2
family with sequence similarity 13, member C	FAM13C	Hs.607594	2.88	7.2E-03	chr10q21.1
family with sequence similarity 172, member A	FAM172A	Hs.600086	2.84	6.8E-03	chr5q15
229544_at		Hs.167087 ///	2.73	2.5E-03	
chromosome 7 open reading frame 41	C7orf41	Hs.200100	2.69	5.2E-03	chr7p15.1
zinc finger CCCH-type, antiviral 1	ZC3HAV1	Hs.133512	2.66	6.0E-03	chr7q34
nuclear transcription factor Y, alpha	NFYA	Hs.10441	2.62	2.6E-02	chr6p21.3
chromodomain helicase DNA binding protein 2	CHD2	Hs.220864	2.62	1.7E-02	chr15q26
myosin VB	MYO5B	Hs.200136	2.62	1.2E-02	chr18q21
225725_at		Hs.371609	2.60	6.7E-03	
228120_at		Hs.656677	2.56	8.5E-03	
hydroxysteroid (17-beta) dehydrogenase 6 homolog (mouse)	HSD17B6	Hs.524513	2.52	1.3E-02	chr12q13

SM-Table 2: Genes Significantly Downregulated (>2 fold, $p<0.05$) in HTM Cells Phagocytically Challenged to E. coli Under Physiological Conditions

Gene Title	Gene Symbol	UniGene ID	Fold	PValue	Chromosomal Location
collagen, type XI, alpha 1	COL11A1	Hs.523446	2.51	1.0E-02	chr1p21
sodium channel, voltage-gated, type II, alpha subunit	SCN2A	Hs.93485	2.50	8.4E-03	chr2q23-q24
5'-nucleotidase domain containing 2	NT5DC2	Hs.84753	2.46	7.6E-03	chr3p21.1
235355_at		Hs.609017	2.45	7.8E-03	
fibrillin 2	FBN2	Hs.519294	2.44	1.9E-02	chr5q23-q31
aldehyde dehydrogenase 1 family, member A1	ALDH1A1	Hs.76392	2.44	3.6E-02	chr9q21.13
scinderin	SCIN	Hs.655515	2.43	1.1E-02	chr7p21.3
protein phosphatase 1E (PP2C domain containing)	PPM1E	Hs.245044	2.39	9.1E-03	chr17q22
vasohibin 2	VASH2	Hs.96885	2.35	1.0E-02	chr1q32.3
229004_at		Hs.534221	2.34	4.2E-03	
endomucin	EMCN	Hs.152913	2.33	1.4E-02	chr4q23
229569_at		Hs.49329	2.32	2.7E-03	
ankyrin repeat domain 6	ANKRD6	Hs.702213	2.32	2.7E-03	chr6q14.2-q16.1
ribosomal protein S20	RPS20	Hs.8102	2.31	1.1E-02	chr8q12
240747_at		Hs.667630	2.30	3.4E-03	
metallophosphoesterase domain containing 2	MPPED2	Hs.289795	2.29	2.3E-02	chr11p13
241560_at		Hs.164225	2.28	1.3E-02	
ArfGAP with RhoGAP domain, ankyrin repeat and PH domain 2	ARAP2	Hs.479451	2.27	1.1E-02	chr4p14
213675_at		Hs.432914	2.27	4.6E-03	
cyclin-dependent kinase inhibitor 1C (p57, Kip2)	CDKN1C	Hs.106070	2.27	4.8E-03	chr11p15.5
melanocortin 2 receptor accessory protein 2	MRAP2	Hs.370055	2.27	5.4E-03	chr6q14.3
potassium voltage-gated channel, delayed-rectifier, subfamily S, member 2	KCNS2	Hs.388045	2.25	1.0E-02	chr8q22
protease, serine, 35	PRSS35	Hs.98381	2.25	2.1E-03	chr6q14.2
242899_at		Hs.120633	2.24	3.1E-02	
transmembrane protein 37	TMEM37	Hs.26216	2.24	2.9E-02	chr2q14.2
autism susceptibility candidate 2	AUTS2	Hs.654801	2.23	3.4E-03	chr7q11.22
growth hormone receptor	GHR	Hs.125180	2.22	7.8E-03	chr5p13-p12
WAP four-disulfide core domain 1	WFDC1	Hs.36688	2.22	2.2E-03	chr16q24.3
zinc finger, matrin type 3	ZMAT3	Hs.386299	2.22	4.7E-03	chr3q26.3-q27
protocadherin beta 6	PCDHB6	Hs.283085	2.21	3.6E-02	chr5q31
septin 11	SEPT11	Hs.128199	2.21	3.2E-02	chr4q21.1
KIAA0114	KIAA0114	Hs.17719	2.20	3.0E-03	chr4q12
RALBP1 associated Eps domain containing 2	REPS2	Hs.186810	2.19	1.5E-02	chrXp22.2

SM-Table 2: Genes Significantly Downregulated (>2 fold, p<0.05) in HTM Cells Phagocytically Challenged to E. coli Under Physiological Conditions

Gene Title	Gene Symbol	UniGene ID	Fold	PValue	Chromosomal Location
glutaminase	GLS	Hs.116448	2.19	6.0E-03	chr2q32-q34
doublecortin-like kinase 1	DCLK1	Hs.507755	2.19	3.1E-02	chr13q13
237400_at		Hs.656689	2.19	3.9E-03	
matrix metallopeptidase 16 (membrane-inserted)	MMP16	Hs.546267	2.19	1.4E-02	chr8q21.3
Tetraspanin 18	TSPAN18	Hs.385634	2.19	3.4E-03	chr11p11.2
jagged 1 (Alagille syndrome)	JAG1	Hs.714736	2.18	2.4E-02	chr20p12.1-p11.23
selenoprotein P, plasma, 1	SEPP1	Hs.275775	2.18	1.3E-02	chr5q31
osteoglycin	OGN	Hs.109439	2.18	2.6E-03	chr9q22
215306_at		Hs.660870	2.17	4.1E-03	
229072_at		Hs.594773	2.16	1.2E-02	
SLIT and NTRK-like family, member 6	SLITRK6	Hs.525105	2.16	7.8E-03	chr13q31.1
hypothetical LOC730101	LOC730101	Hs.7921	2.15	1.2E-02	chr6p12.1
ring finger protein 144A	RNF144A	Hs.22146	2.15	3.4E-03	chr2p25.2-p25.1
transmembrane protein 119	TMEM119	Hs.449718	2.14	4.0E-03	chr12q24.11
small nuclear ribonucleoprotein polypeptide N	SNRPN	Hs.592473	2.14	4.1E-02	chr15q11.2
aldehyde dehydrogenase 5 family, member A1	ALDH5A1	Hs.371723	2.14	1.3E-02	chr6p22.2-p22.3
osteomodulin	OMD	Hs.94070	2.13	5.2E-03	chr9q22.31
sema domain, transmembrane domain (TM), and cytoplasmic domain, (semaphorin) 6A	SEMA6A	Hs.156967	2.13	2.3E-02	chr5q23.1
sodium leak channel, non-selective	NALCN	Hs.525146	2.13	2.8E-02	chr13q32.3
snail homolog 2 (Drosophila)	SNAI2	Hs.360174	2.12	2.6E-03	chr8q11
TAF9B RNA polymerase II, TATA box binding protein (TBP)-associated factor, 31kDa	TAF9B	Hs.592248	2.11	2.5E-02	chrXq13.1-q21.1
matrix Gla protein	MGP	Hs.365706	2.11	4.0E-02	chr12p13.1-p12.3
family with sequence similarity 129, member A	FAM129A	Hs.518662	2.10	2.9E-02	chr1q25
sarcoglycan, gamma (35kDa dystrophin-associated glycoprotein)	SGCG	Hs.37167	2.09	2.9E-03	chr13q12
1557275_a_at		Hs.547494	2.09	3.7E-02	
ankyrin repeat domain 50	ANKRD50	Hs.480694	2.09	1.9E-02	chr4q28.1
236297_at		Hs.585479	2.08	3.2E-03	
phospholipase C, epsilon 1	PLCE1	Hs.655033	2.08	1.7E-02	chr10q23
leucine-rich, glioma inactivated 1	LGI1	Hs.533670	2.08	1.2E-02	chr10q24
corin, serine peptidase	CORIN	Hs.518618	2.07	4.0E-03	chr4p13-p12
229823_at		Hs.709812	2.07	2.6E-02	
Ras association (RalGDS/AF-6) domain family member 2	RASSF2	Hs.631504	2.06	3.7E-03	chr20pter-p12.1

SM-Table 2: Genes Significantly Downregulated (>2 fold, p<0.05) in HTM Cells Phagocytically Challenged to E. coli Under Physiological Conditions

Gene Title	Gene Symbol	UniGene ID	Fold	PValue	Chromosomal Location
transmembrane and coiled-coil domain family 3	TMCC3	Hs.370410	2.06	2.3E-02	chr12q22
synaptopodin 2	SYNPO2	Hs.655519	2.04	1.7E-02	chr4q26
family with sequence similarity 149, member A	FAM149A	Hs.357025	2.01	8.6E-03	chr4q35.2
collagen, type VIII, alpha 2	COL8A2	Hs.353001	2.00	6.2E-03	chr1p34.2
B-cell CLL/lymphoma 11A (zinc finger protein)	BCL11A	Hs.370549	2.00	1.1E-02	chr2p16.1
retinol binding protein 1, cellular	RBP1	Hs.529571	2.00	3.5E-03	chr3q23
potassium intermediate/small conductance calcium-activated channel, subfamily N, member 2	KCNN2	Hs.98280	2.00	2.8E-02	chr5q22.3
