# Supplementary material for: Up-Regulated Expression of Extracellular Matrix Remodeling Genes in Phagocytically Challenged Trabecular Meshwork Cells
Source: PLoS One. 2012 Apr 18;7(4):e34792. doi: 10.1371/journal.pone.0034792 (PMC3329506; doi:10.1371/journal.pone.0034792)
Supplement: Table S3 — List of genes significantly upregulated (>1.5 fold, p<0.05) in HTM Cells phagocytically challenged to pigment particles under physiological conditions. Confluent cultures of HTM cells were grown for two weeks under physiological 5% O2 atmosphere, and then phagocytically challenged to pigment particles. Changes in gene expression at day 3 post-phagocytic challenge were evaluated by gene array using Affymetrix Human Genome U133 Plus 2.0 chips, and analyzed by Genespring Software. (PDF) [file pone.0034792.s004.pdf]

**SM-Table 3: Genes Significantly Upregulated (>1.5 fold, p<0.05) in HTM Cells Phagocytically Challenged to Pigment Under Physiological Conditions**

| Gene Title | Gene Symbol | UniGene ID | Fold | PValue | Chromosomal Location |
|---|---|---|---|---|---|
| interleukin 33 | IL33 | Hs.348390 | 2.16 | 4.3E-03 | chr9p24.1 |
| matrix metallopeptidase 3 (stromelysin 1, progelatinase) | MMP3 | Hs.375129 | 2.09 | 6.0E-03 | chr11q22.3 |
| matrix metallopeptidase 1 (interstitial collagenase) | MMP1 | Hs.83169 | 1.98 | 5.9E-03 | chr11q22.3 |
| deiodinase, iodothyronine, type II | DIO2 | Hs.202354 | 1.92 | 6.0E-03 | chr14q24.2-q24.3 |
| tumor necrosis factor (ligand) superfamily, member 11 | TNFSF11 | Hs.333791 | 1.87 | 9.1E-03 | chr13q14 |
| tissue factor pathway inhibitor 2 | TFPI2 | Hs.438231 | 1.81 | 1.2E-02 | chr7q22 |
| 230746_s_at | | Hs.713064 | 1.80 | 2.8E-03 | |
| kynureninase (L-kynurenine hydrolase) | KYNU | Hs.470126 | 1.75 | 1.3E-02 | chr2q22.2 |
| Growth arrest-specific 2 like 3 | GAS2L3 | Hs.20575 | 1.63 | 2.5E-02 | chr12q23.1 |
| discs, large (Drosophila) homolog-associated protein 5 | DLGAP5 | Hs.77695 | 1.62 | 1.3E-02 | chr14q22.3 |
| cyclin B1 | CCNB1 | Hs.23960 | 1.61 | 2.1E-02 | chr5q12 |
| centromere protein A | CENPA | Hs.1594 | 1.59 | 2.1E-02 | chr2p24-p21 |
| kinesin family member 20A | KIF20A | Hs.718626 | 1.59 | 1.9E-02 | chr5q31 |
| forkhead box Q1 | FOXQ1 | Hs.591352 | 1.59 | 2.8E-02 | chr6p25 |
| UDP-N-acetyl-alpha-D-galactosamine:polypeptide N-acetylgalactosaminyltransferase-like 2 | GALNTL2 | Hs.411308 | 1.59 | 2.2E-02 | chr3p24.3 |
| superoxide dismutase 2, mitochondrial | SOD2 | Hs.487046 | 1.56 | 6.0E-03 | chr6q25.3 |
| NIMA (never in mitosis gene a)-related kinase 2 | NEK2 | Hs.153704 | 1.56 | 9.2E-03 | chr1q32.2-q41 |
| six transmembrane epithelial antigen of the prostate 1 | STEAP1 | Hs.61635 | 1.53 | 5.3E-03 | chr7q21 |
| hyaluronan-mediated motility receptor (RHAMM) | HMMR | Hs.72550 | 1.52 | 4.3E-02 | chr5q33.2-qter |
| family with sequence similarity 83, member D | FAM83D | Hs.472716 | 1.52 | 1.7E-02 | chr20q11.22-q12 |
| chemokine (C-X-C motif) ligand 6 (granulocyte chemotactic protein 2) | CXCL6 | Hs.164021 | 1.51 | 1.4E-02 | chr4q21 |
| stanniocalcin 1 | STC1 | Hs.25590 | 1.51 | 2.8E-03 | chr8p21-p11.2 |
| solute carrier family 39 (zinc transporter), member 8 | SLC39A8 | Hs.288034 | 1.51 | 8.0E-03 | chr4q22-q24 |
| coxsackie virus and adenovirus receptor | CXADR | Hs.634837 | 1.51 | 1.9E-02 | chr21q21.1 |
| SPC25, NDC80 kinetochore complex component, homolog (S. cerevisiae) | SPC25 | Hs.421956 | 1.50 | 1.5E-02 | chr2q24.3 |
| TTK protein kinase | TTK | Hs.169840 | 1.50 | 2.1E-02 | chr6q13-q21 |
