# Supplementary material for: Up-Regulated Expression of Extracellular Matrix Remodeling Genes in Phagocytically Challenged Trabecular Meshwork Cells
Source: PLoS One. 2012 Apr 18;7(4):e34792. doi: 10.1371/journal.pone.0034792 (PMC3329506; doi:10.1371/journal.pone.0034792)
Supplement: Table S4 — List of genes significantly downregulated (>1.5 fold, p<0.05) in HTM Cells phagocytically challenged to pigment particles under physiological conditions. Confluent cultures of HTM cells were grown for two weeks under physiological 5% O2 atmosphere, and then phagocytically challenged to pigment particles. Changes in gene expression at day 3 post-phagocytic challenge were evaluated by gene array using Affymetrix Human Genome U133 Plus 2.0 chips, and analyzed by Genespring Software. (PDF) [file pone.0034792.s005.pdf]

SM-Table 4: Genes Significantly Downregulated (>1.5 fold, p<0.05) in HTM Cells Phagocytically Challenged to Pigment Under Physiological Conditions

Gene Title	Gene Symbol	UniGene ID	Fold	PValue	Chromosomal Location
ankyrin repeat domain 6	ANKRD6	Hs.702213	1.82	2.7E-02	chr6q14.2-q16.1
Tetraspanin 18	TSPAN18	Hs.385634	1.77	6.0E-03	chr11p11.2
tetraspanin 2	TSPAN2	Hs.310458	1.71	2.2E-02	chr1p13.2
244826_at		Hs.653282	1.69	2.9E-02	
lysosomal-associated membrane protein 3	LAMP3	Hs.518448	1.66	8.9E-03	chr3q26.3-q27
H19, imprinted maternally expressed transcript (non-protein coding)	H19	Hs.533566	1.63	5.9E-03	chr11p15.5
oxytocin receptor	OXTR	Hs.2820	1.63	3.1E-02	chr3p25
229795_at		Hs.48945	1.62	1.0E-02	
fibroblast growth factor receptor 3	FGFR3	Hs.1420	1.61	1.9E-02	chr4p16.3
WAP four-disulfide core domain 1	WFDC1	Hs.36688	1.58	4.3E-03	chr16q24.3
growth arrest-specific 5 (non-protein coding)	GAS5	Hs.656411	1.53	3.8E-02	chr1q25.1
ArfGAP with RhoGAP domain, ankyrin repeat and PH domain 2	ARAP2	Hs.479451	1.52	2.0E-02	chr4p14
hydroxysteroid (17-beta) dehydrogenase 6 homolog (mouse)	HSD17B6	Hs.524513	1.51	2.3E-02	chr12q13
aquaporin 1 (Colton blood group)	AQP1	Hs.76152	1.51	1.2E-02	chr7p14
