# Supplementary material for: Up-Regulated Expression of Extracellular Matrix Remodeling Genes in Phagocytically Challenged Trabecular Meshwork Cells
Source: PLoS One. 2012 Apr 18;7(4):e34792. doi: 10.1371/journal.pone.0034792 (PMC3329506; doi:10.1371/journal.pone.0034792)
Supplement: Table S5 — Quantitative real-time PCR confirmation of selected genes with differential expression in phagocytically challenged human and porcine TM cells under physiological and oxidative stress conditions. The expression levels were calculated using the formula 2-ΔCt, where ΔCt = Ctgene-Ct average housekeeping. β-Actin, GAPDH, and HPRT1 served as internal standard for normalization. Values represent mean ± SD, t-test, n = 3. (*) compares phagocytically challenged versus control cultures; (#) compares oxidatively stressed versus cultures grown under physiological conditions. (PDF) [file pone.0034792.s006.pdf]

SM-Table 5: Summary of the Expression Level and pValues Obtained by qPCR

			Control		E. coli			Pigment		
			2^-DCt	(#) pValue	2^-DCt	(*) pValue	(#) pValue	2^-DCt	(*) pValue	(#) pValue
5% O2	***Human***	***MMP1***	1.96±0.029		14.37±0.034	<0.0001		5.05±0.018	<0.0001	
		MMP3	0.163±0.019		1.483±0.002	<0.0001		0.419±0.001	<0.0001	
		TFPI2	0.099±0.02		0.657±0.082	0.0003		0.2±0.042	0.0198	
		LAMC2	0.149±0.03		0.189±0.031	>0.05		0.177±0.03	>0.05	
		TNFSF11	0.078±0.03		0.137±0.072	0.048		0.15±0.012	0.0182	
		EDN3	0.471±0.091		0.044±0.007	0.0013		0.351±0.054	>0.05	
	Porcine	***MMP1***	2.32±1.105		304.5±79.185	<0.0001		12.31±1.84	<0.0001	
		MMP3	0.029±0.015		52.305±5.034	<0.0001		0.073±0.001	0.007	
		TNFSF11	0.087±0.03		0.222±0.039	0.009		0.032±0.004	0.0346	
40% O2	***Human***	***MMP1***	1.948±0.003	>0.05	7.514±0.331	<0.0001	<0.0001	3.982±0.254	>0.05	0.0019
		MMP3	0.257±0.045	>0.05	1.375±0.018	<0.0001	>0.05	0.376±0.338	0.0002	>0.05
		TFPI2	0.188±0.125	>0.05	0.859±0.057	0.0011	0.0248	0.212±0.013	>0.05	>0.05
		LAMC2	0.08±0.006	0.0174	0.123±0.018	0.0172	0.0332	0.19±0.042	>0.05	>0.05
		TNFSF11	0.035±0.003	>0.05	0.06±0.002	0.0003	>0.05	0.067±0.002	0.0001	0.0003
		EDN3	0.091±0.068	0.0237	0.007±0.005	0.0065	>0.05	0.054±0.01	>0.05	0.0073
	Porcine	***MMP1***	21.440±5.778	<0.0001	316.204±34	<0.0001	>0.05	35.711±3.959	0.0243	0.0007
		MMP3	0.065±0.006	0.0182	50.436±16.739	<0.0001	>0.05	0.156±0.068	>0.05	>0.05
		TNFSF11	0.02±0.007	0.0197	0.619±0.168	0.0035	0.0163	0.009±0.002	>0.05	0.0009
