# Supplementary material for: Up-Regulated Expression of Extracellular Matrix Remodeling Genes in Phagocytically Challenged Trabecular Meshwork Cells
Source: PLoS One. 2012 Apr 18;7(4):e34792. doi: 10.1371/journal.pone.0034792 (PMC3329506; doi:10.1371/journal.pone.0034792)
Supplement: Table S6 — List of the genes significantly upregulated (>2 fold, p<0.05) in HTM Cells phagocytically challenged to E. coli under oxidative stress conditions. Confluent cultures of HTM cells were grown for two weeks under oxidative 40% O2 atmosphere, and then phagocytically challenged to E. coli bioparticles. Changes in gene expression at day 3 post-phagocytic challenge were evaluated by gene array using Affymetrix Human Genome U133 Plus 2.0 chips, and analyzed by Genespring Software. (PDF) [file pone.0034792.s007.pdf]

SM-Table 6: Genes Significantly Upregulated (>2 fold, p<0.05) in HTM Cells Phagocytically Challenged to E. coli Under Oxidative Stress Conditions

Gene Title	Gene Symbol	UniGene ID	Fold	PValue	Chromosomal Location
chemokine (C-X-C motif) ligand 11	CXCL11	Hs.632592	98.99	4.86E-07	chr4q21.2
chemokine (C-X-C motif) ligand 5	CXCL5	Hs.89714	67.17	1.93E-06	chr4q12-q13
myxovirus (influenza virus) resistance 1, interferon-inducible protein p78 (mouse)	MX1	Hs.517307	59.26	1.70E-06	chr21q22.3
interferon-induced protein 44-like	IFI44L	Hs.389724	50.42	1.48E-06	chr1p31.1
chemokine (C-X-C motif) ligand 2	CXCL2	Hs.75765	46.84	1.48E-06	chr4q21
chemokine (C-X-C motif) ligand 3	CXCL3	Hs.89690	41.74	2.15E-06	chr4q21
interferon, alpha-inducible protein 27	IFI27	Hs.532634	33.35	2.50E-06	chr14q32
radical S-adenosyl methionine domain containing 2	RSAD2	Hs.17518	30.30	2.21E-06	chr2p25.2
2',5'-oligoadenylate synthetase 1, 40/46kDa	OAS1	Hs.524760	30.05	4.78E-06	chr12q24.1
ISG15 ubiquitin-like modifier	ISG15	Hs.458485	28.82	1.08E-06	chr1p36.33
chemokine (C-C motif) ligand 8	CCL8	Hs.271387	26.82	7.28E-06	chr17q11.2
2'-5'-oligoadenylate synthetase 2, 69/71kDa	OAS2	Hs.414332	20.48	1.79E-06	chr12q24.2
cytidine monophosphate (UMP-CMP) kinase 2, mitochondrial	CMPK2	Hs.7155	19.80	5.48E-06	chr2p25.2
chemokine (C-X-C motif) ligand 1 (melanoma growth stimulating activity, alpha)	CXCL1	Hs.789	19.67	3.43E-06	chr4q21
superoxide dismutase 2, mitochondrial	SOD2	Hs.487046	18.82	3.81E-06	chr6q25.3
hect domain and RLD 6	HERC6	Hs.529317	18.63	1.48E-06	chr4q22.1
chemokine (C-C motif) ligand 2	CCL2	Hs.303649	18.60	4.76E-06	chr17q11.2-q12
bone marrow stromal cell antigen 2	BST2	Hs.118110	17.23	4.86E-07	chr19p13.2
interleukin 6 (interferon, beta 2)	IL6	Hs.654458	16.41	1.48E-06	chr7p21
chemokine (C-C motif) ligand 7	CCL7	Hs.251526	16.12	1.65E-06	chr17q11.2-q12
interferon, alpha-inducible protein 6	IFI6	Hs.523847	15.14	5.05E-06	chr1p35
chromosome 15 open reading frame 48	C15orf48	Hs.112242	15.02	7.04E-06	chr15q21.1
chemokine (C-C motif) ligand 20	CCL20	Hs.75498	13.97	7.28E-06	chr2q33-q37
tumor necrosis factor, alpha-induced protein 3	TNFAIP3	Hs.211600	12.67	1.48E-06	chr6q23
cathepsin S	CTSS	Hs.181301	12.52	8.40E-06	chr1q21
interleukin 33	IL33	Hs.348390	12.23	7.04E-06	chr9p24.1
myxovirus (influenza virus) resistance 2 (mouse)	MX2	Hs.926	11.66	4.59E-06	chr21q22.3
nuclear factor of kappa light polypeptide gene enhancer in B-cells inhibitor, zeta	NFKBIZ	Hs.319171	11.40	1.30E-05	chr3p12-q12
interferon-induced protein with tetratricopeptide repeats 3	IFIT3	Hs.714337	11.34	1.86E-06	chr10q24
interferon-induced protein with tetratricopeptide repeats 1	IFIT1	Hs.20315	11.16	1.70E-06	chr10q25-q26
complement component 2 /// complement factor B	C2 /// CFB	Hs.408903	11.09	1.65E-06	chr6p21.3
interferon induced with helicase C domain 1	IFIH1	Hs.163173	10.68	1.48E-06	chr2q24

SM-Table 6: Genes Significantly Upregulated (>2 fold, p<0.05) in HTM Cells Phagocytically Challenged to E. coli Under Oxidative Stress Conditions

Gene Title	Gene Symbol	UniGene ID	Fold	PValue	Chromosomal Location
interleukin 8	IL8	Hs.624	10.06	2.13E-05	chr4q13-q21
solute carrier family 39 (zinc transporter), member 8	SLC39A8	Hs.288034	9.79	7.28E-06	chr4q22-q24
complement component 3	C3	Hs.529053	9.44	2.38E-05	chr19p13.3-p13.2
matrix metallopeptidase 12 (macrophage elastase)	MMP12	Hs.1695	9.41	1.65E-06	chr11q22.3
Ets homologous factor	EHF	Hs.653859	8.50	1.48E-06	chr11p12
chemokine (C-X-C motif) ligand 6 (granulocyte chemotactic protein 2)	CXCL6	Hs.164021	8.38	3.58E-05	chr4q21
tissue factor pathway inhibitor 2	TFPI2	Hs.438231	8.17	2.39E-05	chr7q22
mitogen-activated protein kinase kinase kinase 8	MAP3K8	Hs.432453	8.01	3.20E-05	chr10p11.23
		Hs.660221	7.86	1.26E-06	
		Hs.713064	7.66	6.83E-07	
stanniocalcin 1	STC1	Hs.25590	7.54	1.82E-06	chr8p21-p11.2
intercellular adhesion molecule 1	ICAM1	Hs.643447	7.36	4.35E-05	chr19p13.3-p13.2
2'-5'-oligoadenylate synthetase 3, 100kDa	OAS3	Hs.528634	7.31	1.17E-05	chr12q24.2
serpin peptidase inhibitor, clade B (ovalbumin), member 2	SERPINB2	Hs.594481	7.30	7.58E-06	chr18q21.3
tryptophan 2,3-dioxygenase	TDO2	Hs.183671	7.25	7.28E-06	chr4q31-q32
vascular cell adhesion molecule 1	VCAM1	Hs.109225	7.12	1.08E-06	chr1p32-p31
chemokine (C-X-C motif) ligand 10	CXCL10	Hs.632586	7.10	1.70E-06	chr4q21
matrix metallopeptidase 3 (stromelysin 1, progelatinase)	MMP3	Hs.375129	6.99	6.17E-06	chr11q22.3
baculoviral IAP repeat-containing 3	BIRC3	Hs.127799	6.84	2.80E-06	chr11q22
		Hs.660221	6.84	2.15E-06	
myocardin	MYOCD	Hs.567641	6.74	2.10E-05	chr17p11.2
		Hs.660221	6.36	1.48E-06	
SAM domain and HD domain 1	SAMHD1	Hs.580681	6.25	2.00E-05	chr20pter-q12
XIAP associated factor 1	XAF1	Hs.441975	5.98	1.70E-06	chr17p13.2
hydroxysteroid (11-beta) dehydrogenase 1	HSD11B1	Hs.195040	5.89	1.48E-06	chr1q32-q41
		Hs.657330	5.66	1.13E-03	
tumor necrosis factor, alpha-induced protein 6	TNFAIP6	Hs.437322	5.56	4.25E-07	chr2q23.3
transient receptor potential cation channel, subfamily A, member 1	TRPA1	Hs.716816	5.48	2.97E-06	chr8q13
interleukin-1 receptor-associated kinase 2	IRAK2	Hs.449207	5.33	1.69E-05	chr3p25.3
six transmembrane epithelial antigen of the prostate 1	STEAP1	Hs.61635	5.33	2.51E-06	chr7q21
GTP cyclohydrolase 1	GCH1	Hs.86724	5.30	3.73E-06	chr14q22.1-q22.2
hypothetical protein LOC387763	LOC387763	Hs.714890	5.26	5.10E-05	chr11p11.2
bradykinin receptor B1	BDKRB1	Hs.525572	5.24	7.28E-06	chr14q32.1-q32.2

SM-Table 6: Genes Significantly Upregulated (>2 fold, p<0.05) in HTM Cells Phagocytically Challenged to E. coli Under Oxidative Stress Conditions

Gene Title	Gene Symbol	UniGene ID	Fold	PValue	Chromosomal Location
hect domain and RLD 5	HERC5	Hs.26663	5.21	8.79E-06	chr4q22.1
kynureninase (L-kynurenine hydrolase)	KYNU	Hs.470126	5.12	3.24E-05	chr2q22.2
tripartite motif-containing 14	TRIM14	Hs.575631	5.10	2.52E-06	chr9q22.33
chemokine (C-C motif) ligand 5	CCL5	Hs.514821	5.09	1.28E-05	chr17q11.2-q12
metallothionein 1M	MT1M	Hs.647370	5.08	2.94E-05	chr16q13
XIAP associated factor 1	XAF1	Hs.441975	5.05	2.45E-07	chr17p13.2
prostaglandin-endoperoxide synthase 2 (prostaglandin G/H synthase and cyclooxygenase)	PTGS2	Hs.196384	5.04	3.24E-06	chr1q25.2-q25.3
DEAD (Asp-Glu-Ala-Asp) box polypeptide 58	DDX58	Hs.190622	4.95	3.12E-05	chr9p12
nicotinamide phosphoribosyltransferase	NAMPT	Hs.489615	4.73	5.36E-07	chr7q22.2
cholesterol 25-hydroxylase	CH25H	Hs.47357	4.70	1.48E-06	chr10q23
tumor necrosis factor, alpha-induced protein 2	TNFAIP2	Hs.525607	4.57	2.70E-05	chr14q32
nuclear factor of kappa light polypeptide gene enhancer in B-cells inhibitor, alpha	NFKBIA	Hs.81328	4.53	1.48E-06	chr14q13
interferon-induced protein 44	IFI44	Hs.82316	4.53	1.79E-06	chr1p31.1
interferon regulatory factor 7	IRF7	Hs.166120	4.46	2.07E-06	chr11p15.5
interleukin 11	IL11	Hs.467304	4.33	2.00E-05	chr19q13.3-q13.4
damage-regulated autophagy modulator	DRAM	Hs.525634	4.27	1.86E-06	chr12q23.2
interferon-induced protein with tetratricopeptide repeats 2	IFIT2	Hs.437609	4.19	1.94E-05	chr10q23-q25
signal transducer and activator of transcription 1, 91kDa	STAT1	Hs.715518	4.19	1.93E-06	chr2q32.2
leukemia inhibitory factor (cholinergic differentiation factor)	LIF	Hs.2250	4.13	7.29E-06	chr22q12.2
interferon-induced protein 35	IFI35	Hs.632258	4.07	2.31E-05	chr17q21
chromosome 8 open reading frame 4	C8orf4	Hs.591849	4.05	1.48E-06	chr8p11.2
adenosine monophosphate deaminase (isoform E)	AMPD3	Hs.501890	3.92	3.71E-06	chr11p15
deiodinase, iodothyronine, type II	DIO2	Hs.202354	3.82	1.61E-05	chr14q24.2-q24.3
Norrie disease (pseudoglioma)	NDP	Hs.522615	3.81	7.53E-04	chrXp11.4
zinc finger CCCH-type containing 12A	ZC3H12A	Hs.656294	3.77	7.57E-06	chr1p34.3
cingulin-like 1	CGNL1	Hs.148989	3.76	3.44E-04	chr15q21.3
bone morphogenetic protein 2	BMP2	Hs.73853	3.75	7.04E-06	chr20p12
angiopoietin-like 1	ANGPTL1	Hs.591474	3.74	2.15E-06	chr1q25.2
tumor necrosis factor (ligand) superfamily, member 13b	TNFSF13B	Hs.525157	3.68	1.70E-06	chr13q32-q34
phospholipid scramblase 1	PLSCR1	Hs.130759	3.64	1.57E-05	chr3q23
endothelial cell-specific molecule 1	ESM1	Hs.129944	3.63	6.89E-03	chr5q11.2
epiregulin	EREG	Hs.115263	3.62	4.13E-04	chr4q13.3
ubiquitin specific peptidase 18	USP18	Hs.38260	3.61	2.75E-05	chr22q11.21

SM-Table 6: Genes Significantly Upregulated (>2 fold, p<0.05) in HTM Cells Phagocytically Challenged to E. coli Under Oxidative Stress Conditions

Gene Title	Gene Symbol	UniGene ID	Fold	PValue	Chromosomal Location
matrix metallopeptidase 1 (interstitial collagenase)	MMP1	Hs.83169	3.60	3.96E-06	chr11q22.3
claudin 1	CLDN1	Hs.439060	3.60	1.65E-05	chr3q28-q29
neuronal cell adhesion molecule	NRCAM	Hs.21422	3.51	4.59E-06	chr7q31.1-q31.2
sterile alpha motif domain containing 9	SAMD9	Hs.65641	3.38	2.13E-06	chr7q21.2
Epithelial stromal interaction 1 (breast)	EPSTI1	Hs.546467	3.37	2.87E-05	chr13q13.3
retinoic acid receptor responder (tazarotene induced) 1	RARRES1	Hs.131269	3.35	3.91E-05	chr3q25.32-q25.33
interleukin-1 receptor-associated kinase 3	IRAK3	Hs.369265	3.27	1.68E-04	chr12q14.3
interleukin 32	IL32	Hs.943	3.24	2.45E-05	chr16p13.3
ABI family, member 3 (NESH) binding protein	ABI3BP	Hs.477015	3.21	5.18E-05	chr3q12
poly (ADP-ribose) polymerase family, member 14	PARP14	Hs.518203	3.14	1.65E-05	chr3q21.1
nuclear factor (erythroid-derived 2)-like 3	NFE2L3	Hs.404741	3.12	4.36E-05	chr7p15-p14
tumor necrosis factor (ligand) superfamily, member 11	TNFSF11	Hs.333791	3.11	1.31E-05	chr13q14
membrane-associated ring finger (C3HC4) 3	MARCH3	Hs.132441	3.08	1.59E-05	chr5q23.2
delta/notch-like EGF repeat containing	DNER	Hs.234074	3.06	2.04E-05	chr2q36.3
Wilms tumor 1 associated protein	WTAP	Hs.446091	3.05	1.82E-05	chr6q25-q27
interferon induced transmembrane protein 1 (9-27)	IFITM1	Hs.458414	2.99	9.12E-06	chr11p15.5
proteasome (prosome, macropain) subunit, beta type, 9 (large multifunctional peptidase 2)	PSMB9	Hs.654585	2.99	1.10E-04	chr6p21.3
v-maf musculoaponeurotic fibrosarcoma oncogene homolog F (avian)	MAFF	Hs.517617	2.99	7.28E-06	chr22q13.1
GTP binding protein overexpressed in skeletal muscle	GEM	Hs.654463	2.98	3.20E-05	chr8q13-q21
family with sequence similarity 65, member C	FAM65C	Hs.372578	2.97	1.59E-05	chr20q13.13
sterile alpha motif domain containing 9-like	SAMD9L	Hs.489118	2.96	8.03E-06	chr7q21.2-q21.3
poly (ADP-ribose) polymerase family, member 12	PARP12	Hs.12646	2.90	4.99E-06	chr7q34
solute carrier family 11 (proton-coupled divalent metal ion transporters), member 2	SLC11A2	Hs.505545	2.87	1.16E-06	chr12q13
		Hs.658454	2.85	1.34E-04	
immediate early response 3	IER3	Hs.591785	2.82	7.70E-06	chr6p21.3
BCL2-related protein A1	BCL2A1	Hs.227817	2.82	1.59E-04	chr15q24.3
zinc finger CCCH-type containing 12C	ZC3H12C	Hs.376289	2.81	9.09E-05	chr11q22.3
tripartite motif-containing 14	TRIM14	Hs.575631	2.79	2.61E-04	chr9q22.33
		Hs.462257	2.75	2.39E-05	
podoplanin	PDPN	Hs.468675	2.75	5.00E-07	chr1p36.21
nuclear factor of kappa light polypeptide gene enhancer in B-cells 2 (p49/p100)	NFKB2	Hs.73090	2.74	1.48E-05	chr10q24
Meis homeobox 1	MEIS1	Hs.526754	2.74	1.39E-04	chr2p14-p13

SM-Table 6: Genes Significantly Upregulated (>2 fold, p<0.05) in HTM Cells Phagocytically Challenged to E. coli Under Oxidative Stress Conditions

Gene Title	Gene Symbol	UniGene ID	Fold	PValue	Chromosomal Location
		Hs.592775	2.73	2.07E-06	
interferon regulatory factor 9	IRF9	Hs.1706	2.71	1.17E-04	chr14q11.2
tumor necrosis factor (ligand) superfamily, member 10	TNFSF10	Hs.478275	2.67	1.06E-04	chr3q26
metallothionein 1X	MT1X	Hs.374950	2.65	7.64E-05	chr16q13
histone cluster 2, H2aa3 /// histone cluster 2, H2aa4	HIST2H2AA3	Hs.530461	2.64	4.94E-04	chr1q21.2
mesenchyme homeobox 1	MEOX1	Hs.438	2.63	1.48E-06	chr17q21
interleukin 7 receptor	IL7R	Hs.635723	2.63	3.83E-04	chr5p13
RAB27B, member RAS oncogene family	RAB27B	Hs.25318	2.62	1.17E-04	chr18q21.2
thymidine phosphorylase	TYMP	Hs.592212	2.62	6.62E-06	chr22q13|22q13.33
signal transducer and activator of transcription 4	STAT4	Hs.80642	2.60	1.01E-04	chr2q32.2-q32.3
G0/G1switch 2	G0S2	Hs.432132	2.59	1.17E-05	chr1q32.2|1q32.2-q4
CCAAT/enhancer binding protein (C/EBP), delta	CEBPD	Hs.440829	2.59	1.48E-04	chr8p11.2-p11.1
leucine aminopeptidase 3	LAP3	Hs.570791	2.59	3.88E-05	chr4p15.32
KIAA0146	KIAA0146	Hs.381058	2.58	1.58E-05	chr8q11.21
pleckstrin homology-like domain, family A, member 1	PHLDA1	Hs.602085	2.58	1.04E-05	chr12q15
metallothionein 1E	MT1E	Hs.534330	2.57	2.53E-04	chr16q13
solute carrier family 7 (cationic amino acid transporter, y+ system), member 2	SLC7A2	Hs.448520	2.57	1.03E-05	chr8p22-p21.3
		Hs.655937	2.56	2.95E-05	
metallothionein 1F	MT1F	Hs.513626	2.54	8.15E-05	chr16q13
cytochrome P450, family 7, subfamily B, polypeptide 1	CYP7B1	Hs.667720	2.54	7.32E-04	chr8q21.3
		Hs.593316	2.53	4.36E-05	
secreted phosphoprotein 1	SPP1	Hs.313	2.53	1.64E-03	chr4q21-q25
carbonic anhydrase XII	CA12	Hs.210995	2.53	6.83E-07	chr15q22
NGFI-A binding protein 1 (EGR1 binding protein 1)	NAB1	Hs.570078	2.53	3.54E-05	chr2q32.3-q33
proteasome (prosome, macropain) activator subunit 2 (PA28 beta)	PSME2	Hs.434081	2.52	1.10E-05	chr14q11.2
hypothetical protein LOC100132999	LOC1001329	Hs.670106	2.52	4.71E-05	chr1q21.1
pigeon homolog (Drosophila)	PION	Hs.186649	2.51	6.88E-05	chr7q11.23
integrin, alpha 2 (CD49B, alpha 2 subunit of VLA-2 receptor)	ITGA2	Hs.482077	2.51	6.26E-05	chr5q23-q31
receptor (chemosensory) transporter protein 4	RTP4	Hs.43388	2.50	7.17E-05	chr3q27.3
R-spondin 3 homolog (Xenopus laevis)	RSPO3	Hs.135254	2.49	4.36E-05	chr6q22.33
similar to WDNM1-like protein	LOC645638	Hs.463652	2.49	3.20E-05	chr17q23.1
solute carrier family 39 (zinc transporter), member 14	SLC39A14	Hs.491232	2.47	1.90E-05	chr8p21.3
F-box protein 32	FBXO32	Hs.403933	2.46	1.90E-04	chr8q24.13

SM-Table 6: Genes Significantly Upregulated (>2 fold, p<0.05) in HTM Cells Phagocytically Challenged to E. coli Under Oxidative Stress Conditions

Gene Title	Gene Symbol	UniGene ID	Fold	PValue	Chromosomal Location
solute carrier family 15, member 3	SLC15A3	Hs.237856	2.44	3.34E-05	chr11q12.2
Pelota homolog (Drosophila)	PELO	Hs.644352	2.44	9.99E-04	chr5q11.2
glycoprotein M6B	GPM6B	Hs.495710	2.43	5.96E-06	chrXp22.2
leucine rich repeat neuronal 3	LRRN3	Hs.3781	2.41	2.32E-04	chr7q31.1
sorbin and SH3 domain containing 2	SORBS2	Hs.655143	2.39	7.20E-06	chr4q35.1
synuclein, alpha interacting protein	SNCAIP	Hs.426463	2.39	5.72E-06	chr5q23.1-q23.3
metallothionein 1H	MT1H	Hs.438462	2.38	7.25E-05	chr16q13
v-rel reticuloendotheliosis viral oncogene homolog B	RELB	Hs.654402	2.38	1.31E-04	chr19q13.32
deltex 3-like (Drosophila)	DTX3L	Hs.518201	2.38	7.58E-06	chr3q21.1
chitinase 3-like 1 (cartilage glycoprotein-39)	CHI3L1	Hs.382202	2.36	2.56E-05	chr1q32.1
v-yes-1 Yamaguchi sarcoma viral related oncogene homolog	LYN	Hs.699154	2.35	1.52E-04	chr8q13
chemokine (C-C motif) receptor-like 1	CCRL1	Hs.310512	2.35	5.01E-05	chr3q22
2'-5'-oligoadenylate synthetase-like	OASL	Hs.118633	2.34	2.39E-04	chr12q24.2
PR domain containing 1, with ZNF domain	PRDM1	Hs.436023	2.34	3.60E-04	chr6q21-q22.1
metallothionein 1G	MT1G	Hs.433391	2.33	5.48E-05	chr16q13
bradykinin receptor B2	BDKRB2	Hs.719484	2.33	1.26E-05	chr14q32.1-q32.2
ubiquitin-conjugating enzyme E2L 6	UBE2L6	Hs.425777	2.31	9.25E-05	chr11q12
ELOVL family member 7, elongation of long chain fatty acids (yeast)	ELOVL7	Hs.274256	2.31	6.59E-03	chr5q12.1
caspase 1, apoptosis-related cysteine peptidase (interleukin 1, beta, convertase)	CASP1	Hs.2490	2.31	2.31E-04	chr11q23
tyrosinase-related protein 1	TYRP1	Hs.270279	2.30	7.91E-04	chr9p23
chromosome 13 open reading frame 33	C13orf33	Hs.646647	2.30	2.94E-05	chr13q12.3
regulator of calcineurin 1	RCAN1	Hs.282326	2.29	2.49E-05	chr21q22.1-q22.2|21
eukaryotic translation initiation factor 2-alpha kinase 2	EIF2AK2	Hs.131431	2.29	5.16E-03	chr2p22-p21
nuclear receptor subfamily 4, group A, member 2	NR4A2	Hs.563344	2.29	1.13E-05	chr2q22-q23
vascular endothelial growth factor C	VEGFC	Hs.435215	2.28	1.65E-06	chr4q34.1-q34.3
phospholipase A2, group IVC (cytosolic, calcium-independent)	PLA2G4C	Hs.631562	2.27	7.27E-04	chr19q13.3
solute carrier family 16, member 3 (monocarboxylic acid transporter 4)	SLC16A3	Hs.500761	2.27	3.03E-05	chr17q25
acyl-CoA synthetase long-chain family member 5	ACSL5	Hs.11638	2.27	3.55E-05	chr10q25.1-q25.2
adenylate kinase 3-like 1	AK3L1	Hs.592601	2.26	1.50E-05	chr1p31.3
guanylate binding protein 1, interferon-inducible, 67kDa	GBP1	Hs.62661	2.26	7.28E-06	chr1p22.2

SM-Table 6: Genes Significantly Upregulated (>2 fold, p<0.05) in HTM Cells Phagocytically Challenged to E. coli Under Oxidative Stress Conditions

Gene Title	Gene Symbol	UniGene ID	Fold	PValue	Chromosomal Location
transporter 1, ATP-binding cassette, sub-family B (MDR/TAP)	TAP1	Hs.352018	2.26	2.45E-05	chr6p21.3
phospholipase A2, group IVA (cytosolic, calcium-dependent)	PLA2G4A	Hs.497200	2.25	1.98E-04	chr1q25
prostaglandin E synthase	PTGES	Hs.146688	2.25	9.44E-05	chr9q34.3
spermatogenesis associated 13	SPATA13	Hs.657121	2.23	2.29E-05	chr13q12.12
metallothionein 1 pseudogene 2	MT1P2	Hs.632513	2.22	1.31E-04	chr1q43
serglycin	SRGN	Hs.1908	2.22	3.51E-05	chr10q22.1
ring finger protein 144B	RNF144B	Hs.148741	2.21	1.08E-05	chr6p22.3
ATPase, class I, type 8B, member 4	ATP8B4	Hs.511311	2.20	1.92E-04	chr15q21.2
spermidine/spermine N1-acetyltransferase 1	SAT1	Hs.28491	2.20	5.29E-04	chrXp22.1
		Hs.546523	2.20	2.94E-05	
metallothionein 2A	MT2A	Hs.647371	2.20	4.77E-05	chr16q13
hyaluronan synthase 2	HAS2	Hs.159226	2.19	1.26E-04	chr8q24.12
poly (ADP-ribose) polymerase family, member 9	PARP9	Hs.518200	2.19	2.94E-05	chr3q21
phospholipase C-like 1	PLCL1	Hs.153322	2.18	1.57E-03	chr2q33
interleukin 1, beta	IL1B	Hs.126256	2.18	9.38E-04	chr2q14
BCL2/adenovirus E1B 19kDa interacting protein 3	BNIP3	Hs.144873	2.18	2.53E-05	chr10q26.3
protein kinase (cAMP-dependent, catalytic) inhibitor beta	PKIB	Hs.719228	2.17	4.49E-04	chr6q22.31
acyl-CoA synthetase long-chain family member 4	ACSL4	Hs.268785	2.16	8.31E-05	chrXq22.3-q23
butyrophilin, subfamily 3, member A2	BTN3A2	Hs.376046	2.16	3.68E-05	chr6p22.1
procollagen-lysine, 2-oxoglutarate 5-dioxygenase 2	PLOD2	Hs.477866	2.15	1.89E-05	chr3q23-q24
UDP-glucose ceramide glucosyltransferase	UGCG	Hs.304249	2.15	4.28E-05	chr9q31
interferon, gamma-inducible protein 30	IFI30	Hs.14623	2.14	2.29E-05	chr19p13.1
family with sequence similarity 20, member A	FAM20A	Hs.268874	2.14	3.12E-05	chr17q24.2
Uncharacterized protein LOC100131897	LOC1001318	Hs.659330	2.14	1.77E-04	chr5q35.1
			2.14	1.71E-04	
			2.13	5.55E-05	
outer dense fiber of sperm tails 3B	ODF3B	Hs.531314	2.13	2.16E-03	chr22q13.33
arylacetamide deacetylase-like 1	AADACL1	Hs.444099	2.12	5.99E-04	chr3q26.31
fibroblast growth factor 10	FGF10	Hs.664499	2.12	1.91E-03	chr5p13-p12
toll-like receptor 3	TLR3	Hs.657724	2.12	4.67E-05	chr4q35
		Hs.23349	2.12	9.53E-03	
dystrobrevin, alpha	DTNA	Hs.643454	2.11	1.71E-04	chr18q12
interleukin 15 receptor, alpha	IL15RA	Hs.524117	2.11	1.05E-04	chr10p15-p14

SM-Table 6: Genes Significantly Upregulated (>2 fold, p<0.05) in HTM Cells Phagocytically Challenged to E. coli Under Oxidative Stress Conditions

Gene Title	Gene Symbol	UniGene ID	Fold	PValue	Chromosomal Location
tumor necrosis factor (ligand) superfamily, member 18	TNFSF18	Hs.248197	2.11	4.77E-05	chr1q23
similar to hCG38149 /// ovostatin /// ovostatin 2	LOC728715 /	Hs.568152	2.11	2.16E-04	chr12p11.21 /// chr1:
oxidized low density lipoprotein (lectin-like) receptor 1	OLR1	Hs.412484	2.10	2.15E-03	chr12p13.2-p12.3
chromosome 7 open reading frame 58	C7orf58	Hs.189652	2.10	1.10E-05	chr7q31.31
SP100 nuclear antigen	SP100	Hs.369056	2.10	4.13E-05	chr2q37.1
		Hs.201600	2.09	3.76E-06	
methylenetetrahydrofolate dehydrogenase (NADP+ dependent) 2-like	MTHFD2L	Hs.479954	2.09	1.06E-05	chr4q13.3
		Hs.28792	2.09	9.96E-07	
endoplasmic reticulum aminopeptidase 2	ERAP2	Hs.482910	2.07	3.94E-05	chr5q15
FYVE, RhoGEF and PH domain containing 4	FGD4	Hs.117835	2.07	1.31E-04	chr12p11.21
tripartite motif-containing 69	TRIM69	Hs.489254	2.06	1.10E-04	chr15q21.1
glycerol kinase	GK	Hs.1466	2.06	9.08E-04	chrXp21.3
interferon stimulated exonuclease gene 20kDa	ISG20	Hs.459265	2.06	1.74E-05	chr15q26
transporter 2, ATP-binding cassette, sub-family B (MDR/TAP)	TAP2	Hs.502	2.06	1.03E-04	chr6p21.3
		Hs.591248	2.06	6.24E-03	
solute carrier family 22, member 23	SLC22A23	Hs.713588	2.06	4.01E-05	chr6p25.2
		Hs.439682	2.05	2.72E-04	
tripartite motif-containing 25	TRIM25	Hs.528952	2.05	7.55E-05	chr17q23.2
transmembrane protein 132B	TMEM132B	Hs.524838	2.05	6.66E-03	chr12q24.31-q24.32
colony stimulating factor 1 (macrophage)	CSF1	Hs.591402	2.04	2.00E-05	chr1p21-p13
dual specificity phosphatase 6	DUSP6	Hs.298654	2.04	6.41E-05	chr12q22-q23
TNFAIP3 interacting protein 1	TNIP1	Hs.355141	2.04	1.08E-04	chr5q32-q33.1
v-myb myeloblastosis viral oncogene homolog (avian)-like 1	MYBL1	Hs.445898	2.04	1.01E-04	chr8q22
mannosidase, alpha, class 1A, member 1	MAN1A1	Hs.102788	2.04	1.93E-04	chr6q22
caspase recruitment domain family, member 16 /// caspase 1, apoptosis-related cysteine peptidase (interleukin 1, beta, convertase)	CARD16 /// C	Hs.348365	2.03	1.75E-04	chr11q23
			2.03	1.34E-04	
gamma-aminobutyric acid (GABA) B receptor, 1 /// ubiquitin D	GABBR1 /// U	Hs.719331	2.03	1.47E-03	chr6p21.3 /// chr6p2
interleukin 18 binding protein	IL18BP	Hs.591967	2.03	2.34E-06	chr11q13
UDP-GlcNAc:betaGal beta-1,3-N-acetylglucosaminyltransferase 5	B3GNT5	Hs.718506	2.02	2.23E-02	chr3q28

SM-Table 6: Genes Significantly Upregulated (>2 fold, p<0.05) in HTM Cells Phagocytically Challenged to E. coli Under Oxidative Stress Conditions

Gene Title	Gene Symbol	UniGene ID	Fold	PValue	Chromosomal Location
junctional adhesion molecule 2	JAM2	Hs.517227	2.02	1.48E-04	chr21q21.2
			2.02	6.29E-03	
chromosome 6 open reading frame 176	C6orf176	Hs.31917	2.01	3.58E-04	chr6q27
zona pellucida glycoprotein 4	ZP4	Hs.136241	2.01	4.66E-05	chr1q43
tumor necrosis factor receptor superfamily, member 11b	TNFRSF11B	Hs.81791	2.01	6.71E-06	chr8q24
histone cluster 2, H2be	HIST2H2BE	Hs.2178	2.01	1.27E-04	chr1q21-q23
		Hs.634153	2.01	2.66E-04	
basic leucine zipper transcription factor, ATF-like 2	BATF2	Hs.124840	2.00	4.44E-03	chr11q13.1
dipeptidyl-peptidase 4	DPP4	Hs.368912	2.00	3.21E-05	chr2q24.3
chromosome 11 open reading frame 17 /// NUAK family, SNF1-like kinase, 2	C11orf17 /// N	Hs.131180	2.00	1.98E-04	chr11p15.3 /// chr1q
