# Supplementary material for: Up-Regulated Expression of Extracellular Matrix Remodeling Genes in Phagocytically Challenged Trabecular Meshwork Cells
Source: PLoS One. 2012 Apr 18;7(4):e34792. doi: 10.1371/journal.pone.0034792 (PMC3329506; doi:10.1371/journal.pone.0034792)
Supplement: Table S7 — List of the genes significantly downregulated (>2 fold, p<0.05) in HTM Cells phagocytically challenged to E. coli under oxidative stress conditions. Confluent cultures of HTM cells were grown for two weeks under oxidative 40% O2 atmosphere, and then phagocytically challenged to E. coli bioparticles. Changes in gene expression at day 3 post-phagocytic challenge were evaluated by gene array using Affymetrix Human Genome U133 Plus 2.0 chips, and analyzed by Genespring Software. (PDF) [file pone.0034792.s008.pdf]

SM-Table 7: Genes Significantly Downregulated (>2 fold, p<0.05) in HTM Cells Phagocytically Challenged to E. coli Under Oxidative Stress Conditions

Gene Title	Gene Symbol	UniGene ID	Fold	PValue	Chromosomal Location
tumor necrosis factor receptor superfamily, member 10d, decoy with truncated death domain	TNFRSF10D	Hs.213467	4.11	2.13E-06	chr8p21
endothelin 3	EDN3	Hs.1408	4.04	2.59E-05	chr20q13.2-q13.3
ribosomal protein L27a	RPL27A	Hs.523463	3.08	2.97E-04	chr11p15
		Hs.26766	2.76	6.94E-05	
ceroid-lipofuscinosis, neuronal 8 (epilepsy, progressive with mental retardation)	CLN8	Hs.127675	2.68	1.29E-05	chr8p23
sclerostin domain containing 1	SOSTDC1	Hs.648106	2.65	3.88E-05	chr7p21.1
AF4/FMR2 family, member 3	AFF3	Hs.444414	2.64	1.05E-04	chr2q11.2-q12
C1q and tumor necrosis factor related protein 7	C1QTNF7	Hs.153714	2.57	6.13E-05	chr4p16-p15
Fibroblast growth factor 18	FGF18	Hs.87191	2.57	2.61E-06	chr5q34
nanos homolog 1 (Drosophila)	NANOS1	Hs.591918	2.52	2.21E-04	chr10q26.11
aldehyde dehydrogenase 1 family, member A1	ALDH1A1	Hs.76392	2.47	9.83E-04	chr9q21.13
aquaporin 1 (Colton blood group)	AQP1	Hs.76152	2.46	1.45E-03	chr7p14
		Hs.554052	2.41	1.39E-02	
DIRAS family, GTP-binding RAS-like 2	DIRAS2	Hs.165636	2.31	1.37E-04	chr9q22.2
5'-nucleotidase domain containing 2	NT5DC2	Hs.84753	2.27	5.56E-05	chr3p21.1
interleukin 17 receptor D	IL17RD	Hs.150725	2.27	9.36E-04	chr3p14.3
cyclin-dependent kinase inhibitor 1C (p57, Kip2)	CDKN1C	Hs.106070	2.23	5.05E-06	chr11p15.5
odd-skipped related 2 (Drosophila)	OSR2	Hs.253247	2.22	8.40E-06	chr8q22.2
nuclear transcription factor Y, alpha	NFYA	Hs.10441	2.19	6.62E-04	chr6p21.3
EPH receptor A3	EPHA3	Hs.123642	2.18	7.27E-06	chr3p11.2
potassium intermediate/small conductance calcium-activated channel, subfamily N, member 2	KCNN2	Hs.98280	2.12	5.53E-04	chr5q22.3
H19, imprinted maternally expressed transcript (non-protein coding)	H19	Hs.533566	2.08	2.21E-06	chr11p15.5
melanocortin 2 receptor accessory protein 2	MRAP2	Hs.370055	2.07	2.44E-04	chr6q14.3
family with sequence similarity 172, member A	FAM172A	Hs.600086	2.06	3.20E-05	chr5q15
small nuclear ribonucleoprotein polypeptide N	SNRPN	Hs.592473	2.05	2.34E-02	chr15q11.2
secretogranin II (chromogranin C)	SCG2	Hs.516726	2.05	5.20E-06	chr2q35-q36
G protein-coupled receptor 37 (endothelin receptor type B-like)	GPR37	Hs.406094	2.03	1.34E-04	chr7q31
carbohydrate (N-acetylglucosamine 6-O) sulfotransferase 6	CHST6	Hs.655622	2.02	2.35E-04	chr16q22
		Hs.534221	2.01	1.48E-04	
hydroxysteroid (17-beta) dehydrogenase 6 homolog (mouse)	HSD17B6	Hs.524513	2.01	1.58E-03	chr12q13
