# Supplementary material for: Up-Regulated Expression of Extracellular Matrix Remodeling Genes in Phagocytically Challenged Trabecular Meshwork Cells
Source: PLoS One. 2012 Apr 18;7(4):e34792. doi: 10.1371/journal.pone.0034792 (PMC3329506; doi:10.1371/journal.pone.0034792)
Supplement: Table S8 — List of the genes significantly upregulated (>2 fold, p<0.05) in HTM Cells phagocytically challenged to pigment under oxidative stress conditions. Confluent cultures of HTM cells were grown for two weeks under oxidative 40% O2 atmosphere, and then phagocytically challenged to pigment particles. Changes in gene expression at day 3 post-phagocytic challenge were evaluated by gene array using Affymetrix Human Genome U133 Plus 2.0 chips, and analyzed by Genespring Software. (PDF) [file pone.0034792.s009.pdf]

SM-Table 8: Genes Significantly Upregulated (>1.5 fold, p<0.05) in HTM Cells Phagocytically Challenged to Pigment Under Oxidative Stress Conditions

Gene Title	Gene Symbol	UniGene ID	Fold	PValue	Chromosomal Location
tumor necrosis factor (ligand) superfamily, member 11	TNFSF11	Hs.333791	2.12	2.9E-05	chr13q14
matrix metallopeptidase 1 (interstitial collagenase)	MMP1	Hs.83169	1.79	1.8E-05	chr11q22.3
matrix metallopeptidase 3 (stromelysin 1, progelatinase)	MMP3	Hs.375129	1.79	1.8E-05	chr11q22.3
annexin A10	ANXA10	Hs.188401	1.63	1.7E-02	chr4q33
family with sequence similarity 40, member B	FAM40B	Hs.489988	1.63	1.2E-02	chr7q32.1
discs, large (Drosophila) homolog-associated protein 5	DLGAP5	Hs.77695	1.61	9.9E-05	chr14q22.3
RAB27B, member RAS oncogene family	RAB27B	Hs.25318	1.61	2.5E-04	chr18q21.2
centromere protein F, 350/400ka (mitosin)	CENPF	Hs.497741	1.61	1.1E-03	chr1q32-q41
deiodinase, iodothyronine, type II	DIO2	Hs.202354	1.59	6.8E-05	chr14q24.2-q24.3
aquaporin 3 (Gill blood group)	AQP3	Hs.234642	1.58	1.1E-03	chr9p13
cyclin B1	CCNB1	Hs.23960	1.56	6.8E-05	chr5q12
heme oxygenase (decycling) 1	HMOX1	Hs.517581	1.56	2.7E-04	chr22q12|22q13.1
kynureninase (L-kynurenine hydrolase)	KYNU	Hs.470126	1.56	6.8E-05	chr2q22.2
UDP-N-acetyl-alpha-D-galactosamine:polypeptide N-acetylgalactosaminyltransferase-like 2	GALNTL2	Hs.411308	1.54	2.1E-03	chr3p24.3
interleukin 13 receptor, alpha 2	IL13RA2	Hs.336046	1.53	2.7E-03	chrXq13.1-q28
prostaglandin-endoperoxide synthase 1 (prostaglandin G/H synthase and cyclooxygenase)	PTGS1	Hs.201978	1.53	2.8E-04	chr9q32-q33.3
cell division cycle 25 homolog C (S. pombe)	CDC25C	Hs.656	1.49	8.5E-04	chr5q31
hect domain and RLD 5	HERC5	Hs.26663	1.49	2.2E-05	chr4q22.1
aquaporin 11	AQP11	Hs.503345	1.48	5.9E-04	chr11q14.1
centromere protein A	CENPA	Hs.1594	1.48	2.5E-04	chr2p24-p21
CD109 molecule	CD109	Hs.399891	1.48	4.9E-02	chr6q13
leucine rich repeat transmembrane neuronal 2	LRRTM2		1.46	4.8E-02	chr5q31.2
laminin, gamma 2	LAMC2	Hs.591484	1.45	1.1E-03	chr1q25-q31
