# Supplementary material for: Up-Regulated Expression of Extracellular Matrix Remodeling Genes in Phagocytically Challenged Trabecular Meshwork Cells
Source: PLoS One. 2012 Apr 18;7(4):e34792. doi: 10.1371/journal.pone.0034792 (PMC3329506; doi:10.1371/journal.pone.0034792)
Supplement: Table S9 — List of the genes significantly downregulated (>2 fold, p<0.05) in HTM Cells phagocytically challenged to pigment under oxidative stress conditions. Confluent cultures of HTM cells were grown for two weeks under oxidative 40% O2 atmosphere, and then phagocytically challenged to pigment particles. Changes in gene expression at day 3 post-phagocytic challenge were evaluated by gene array using Affymetrix Human Genome U133 Plus 2.0 chips, and analyzed by Genespring Software. (PDF) [file pone.0034792.s010.pdf]

SM-Table 9: Genes Significantly Downregulated (>1.5 fold, p<0.05) in HTM Cells Phagocytically Challenged to Pigment Under Oxidative Stress Conditions

Gene Title	Gene Symbol	UniGene ID	Fold	PValue	Chromosomal Location
oxytocin receptor	OXTR	Hs.2820	1.75	5.9E-04	chr3p25
RAS guanyl releasing protein 1 (calcium and DAG-regulated)	RASGRP1	Hs.591127	1.65	6.8E-05	chr15q14
somatostatin receptor 1	SSTR1	Hs.248160	1.62	8.5E-03	chr14q13
potassium intermediate/small conductance calcium-activated channel, subfamily N, member 2	KCNN2	Hs.98280	1.61	1.0E-03	chr5q22.3
family with sequence similarity 35, member A	FAM35A	Hs.500419	1.58	1.7E-02	chr10q23.2
		Hs.411391	1.57	4.8E-03	
mannose-binding lectin (protein A) 1, pseudogene 1	MBL1P1	Hs.102310	1.53	1.7E-02	chr10q22.2-q22.3
actin, alpha 2, smooth muscle, aorta	ACTA2	Hs.500483	1.53	4.7E-04	chr10q23.3
chromosome 8 open reading frame 34	C8orf34	Hs.491941	1.52	1.5E-03	chr8q13
Fms-related tyrosine kinase 1 (vascular endothelial growth factor/vascular permeability factor receptor)	FLT1	Hs.594454	1.52	1.3E-02	
leucine zipper, putative tumor suppressor 1	LZTS1	Hs.521432	1.49	4.8E-02	chr8p22
keratin associated protein 1-5	KRTAP1-5	Hs.534499	1.46	3.4E-04	chr17q12-q21
